# Phage-host interaction in *Pseudomonas aeruginosa* clinical isolates with functional and altered quorum sensing systems

**DOI:** 10.1128/aem.02402-24

**Published:** 2025-03-04

**Authors:** Dandan Li, Na Li, Yu Chen, Yuxuan Yang, Jue Pan, Jiabing Lin, Xiaodong Gao, Rong Bao, Chunmei Zhou, Suzhen Wang, Bijie Hu, Demeng Tan

**Affiliations:** 1 Department of Infectious Diseases, Zhongshan Hospital, Fudan University 92323 , Shanghai, China; 2 Shanghai Institute of Infectious Disease and Biosecurity, Fudan University 12478 , Shanghai, China; 3 Department of Hospital Infection Management, Zhongshan Hospital, Fudan University 92323 , Shanghai, China; 4 Department of Laboratory Medicine, Zhongshan Hospital, Fudan University 92323 , Shanghai, China; 5 Shanghai Public Health Clinical Center, Fudan University 34748 , Shanghai, China; Indiana University Bloomington, Bloomington, Indiana, USA

**Keywords:** bacteriophage therapy, quorum sensing, *Pseudomonas aeruginosa*, phage receptor, phage-host interactions

## Abstract

Quorum sensing (QS) plays a crucial role in regulating key traits, including the upregulation of phage receptors, which leads to heightened phage susceptibility in *Pseudomonas aeruginosa*. As a result, higher cell densities typically increase the risk of phage invasions. This has led to speculation that bacteria may have evolved strategies to counterbalance this increased susceptibility. Additionally, non-synonymous mutations in LasR, the master regulator of QS, are common among cystic fibrosis patients, but the impact of these mutations on phage interactions remains poorly understood. Here, we systematically investigated the role of QS in shaping these interactions using bacterial strains with functional or altered QS systems. In the QS-functional strain ZS-PA-35, disruption of the Las system reduces cell susceptibility to the type IV pili-dependent phage phipa2, delaying bacterial lysis during the early logarithmic growth phase. At high cell densities, Las-induced dormancy further inhibits phage proliferation despite enhanced phage adsorption. Notably, nutrient supplementation fully restores phage proliferation in the strains with a functional Las system. In contrast, the QS-deficient strain ZS-PA-05, carrying a LasR mutation, fails to regulate phage-host interactions via QS. Moreover, our findings reveal that within mixed microbial populations, cells benefit from the presence of closely related kin, which collectively reduce prey density and limit phage-host interaction frequencies under nutrient-rich conditions. These results underscore the flexibility of QS-regulated defense strategies, highlighting their critical role in optimizing bacterial resilience against phage predation, particularly in heterogeneous communities most vulnerable to phages.

**IMPORTANCE:**

Bacteria have developed various strategies to combat phage infection, posing challenges to phage therapy. In this study, we demonstrate that *Pseudomonas aeruginosa* strains with functional or altered quorum sensing (QS) systems may adapt different survival tactics for prolonged coexistence with phages, contingent upon bacterial population dynamics. The dynamics of phage infection highlight the influence of intrinsic heterogeneity mediated by QS, which leads to the emergence of different phage-host outcomes. These variants may arise as a result of coevolutionary processes or coexistence mechanisms of mutational and non-mutational defense strategies. These insights enhance our comprehension of how bacteria shield themselves against phage attacks and further underscore the complexity of such approaches for successful therapeutic interventions.

## INTRODUCTION

*Pseudomonas aeruginosa* plays a significant role in the occurrence of hospital-acquired infections (1). The formation of biofilms under selective pressure is a pivotal factor contributing to its persistence. This, in turn, results in heightened resistance and tolerance to antimicrobials in clinical settings, posing a substantial challenge to patient treatments (2, 3). Thus, there is an urgent demand for effective alternatives. Bacteriophages are viruses with the ability to infect and eliminate bacterial hosts. Their potent lytic capabilities have sparked renewed interest in utilizing them for treating bacterial infections in clinical trials (4). These trials have demonstrated the potential of phage therapy, showing its ability to reduce mortality in certain cases. However, the rapid development of resistance to phages poses challenges, limiting their therapeutic applications (5). Therefore, a comprehensive understanding of complex phage-host interactions is essential for effectively using phages in therapeutic interventions .

The type IV pilus (T4P) serves as a crucial phage receptor and is utilized by various *P. aeruginosa* phages (6, 7). Coevolutionary studies have consistently highlighted mutations in the genes linked to T4P as key determinants driving phage resistance (8–10). For instance, mutants selected by phages exhibit mutations in the *pilT* or *pilB* genes, resulting in increased antibiotic susceptibilities and impaired motility (7). These studies conclusively show that genetic alterations not only lead to the selection of phage-resistant cells but also impose a significant fitness cost, affecting motility, antibiotic resistance, and biofilm formation, which can affect bacterial community succession (11, 12). Hence, the viability of alternative non-mutational defenses in complex microbial communities becomes a question. Among these alternatives, quorum sensing (QS) is a mechanism utilized by bacteria to synchronize gene expression of collective behavior. The Las, Rhl, and *Pseudomonas* quinolone signal (PQS) systems, extensively examined in *P. aeruginosa*, serve as crucial components (13, 14). LasI, the autoinducer (AI) synthase, is responsible for 3-oxo-C12-homoserine lactone (3OC12-HSL) production. This molecule interacts with the LasR receptor, forming a complex that activates certain gene expression. Furthermore, the LasR-3OC12-HSL complex stimulates genes associated with the RhlI/R and PQS systems. RhlI generates the C4-homoserine lactone (C4-HSL), which, upon binding to RhlR, initiates another set of target genes. The third AI, 2-heptyl-3-hydroxy-4-quinolone (PQS), binds to the PqsR receptor acting as a connector between the Las and Rhl systems. Recent discoveries reveal more flexible defense mechanisms, temporarily upregulating phage receptors or activating *cas* gene expression in response to signaling molecules (15–17). Moreover, phage DMS3 carries an anti-QS protein known as Aqs1. This protein effectively sequesters the key transcription factor LasR, hindering its ability to bind to downstream regulatory elements (18). Based on these studies, it is evident that receptor regulation, in addition to host mutational defenses, can circumvent receptor-mediated fitness costs. This allows the host to mount a defense while preserving the cell lineage.

In clinical phage therapy, bacterial population density, varying across infection sites, influences phage treatment efficacy. High-density conditions intensify phage predation pressure, driving host adaptation and coevolution with phages. Genomic analysis reveals that clinical isolates of *P. aeruginosa* frequently carry *lasR* mutations with a fitness advantage, profoundly influencing traits related to social exploitation, including virulence and antibiotic resistance. And these mutants could maintain QS through LasR-independent Rhl system activation (19–22). However, the potential for social cooperative behaviors in phage-host interactions within heterogeneous *P. aeruginosa* populations during chronic infections remains uninvestigated. We speculate that bacteria may adjust their anti-phage strategies depending on the perceived population and neighboring kin, as well as their physiological metabolic status (i.e., less efficient phage amplification), adopting behaviors suitable for either individual or communal survival accordingly.

Our previous study demonstrated that introducing phage phipa2 to the clinical isolate *P. aeruginosa* ZS-PA-35 led to the emergence of surviving populations that did not exhibit mutations in receptor-related genes (7). This finding suggests that bacteria utilize a diverse array of strategies to evade phage predation. Numerous studies have shown that T4P is regulated by QS, and manipulating QS can influence phage susceptibility in strains PAO1 and PA14 (16, 23). In theory, upregulating phage receptor expression in the high-cell-density (HCD) QS mode could result in strong selection pressure of phage receptor-mediated resistance. From an evolutionary perspective, bacteria may develop additional defense strategies to counterbalance the increased phage receptor expression, ensuring the survival of bacterial lineages in dense phage-host environments. However, there is still limited knowledge regarding the clinical isolates and whether their phage-host interactions follow these established regulatory pathways.

Here, we explored phage-host interactions across strains with functional or altered QS systems. In the QS-functional strain ZS-PA-35, deletion of the Las system confers resistance to phage phipa2 infection, thereby reducing phage production, particularly during the early log phase. However, the Las system-induced early dormancy reduces phage proliferation, even though increased adsorption is observed in the HCD cultures. We speculate that by employing these dynamic strategies, bacteria would better adapt to phage predation without incurring a strong fitness cost. Furthermore, in the QS-altered strain ZS-PA-05, the disruption of the Las, Rhl, or PQS systems did not significantly impact phage-host interactions. Notably, under HCD conditions, phage proliferation was more efficient in strain ZS-PA-05, even without the addition of supplementary nutrients. Furthermore, in mixed-species environments, the production of phage phipa2 by the QS-deficient mutant ZS-PA-05 was suppressed by the quorum-competent strain ZS-PA-35 under nutrient-rich conditions. In summary, our findings reveal that *P. aeruginosa* employs diverse anti-phage strategies to ensure its persistence within a heterogeneous population. Importantly, these findings are the first to demonstrate that QS finely regulates phage-host interactions across varying cell densities by coordinating phage receptor expression and phage progeny proliferation in the HCD QS mode, enabling coexistence of both host and phage.

## RESULTS

### Clinical isolate ZS-PA-35 possesses a functional QS system

Building on our initial findings, we first validated the operational status of key QS genes within strain ZS-PA-35. Bioinformatic analysis of strain ZS-PA-35 shows that the *lasI*, *lasR*, and *rhlR* genes are identical to those in PAO1 at the amino acid level, except for two non-synonymous mutations in *rhlI* (S62G and D83E). These mutations, previously identified by the Paczkowski lab in a *lasR* mutant, enable *rhlI* variants to recalibrate autoinducer levels to wild-type (WT) concentrations, thereby restoring virulence phenotypes (24). *P. aeruginosa* has been shown to produce at least two different N-acyl-homoserinelactone (AHL) QS AIs, namely 3OC12-HSL and C4-HSL. To determine whether QS synthases exhibit cell density-dependent activity, we measured AI concentrations at various time points during batch culture growth. As illustrated in Fig. S1A and B, AI concentrations rose markedly in parallel with the increase in culture optical density, particularly during the growth phase from an optical density (OD)_600_ of 0.1 to 1.0. For subsequent assays, we utilized cultures at low cell density (LCD) and HCD corresponding to OD_600_ values of 0.1 and 1.0, respectively, to investigate the effects of QS regulation.

**Fig 1:**
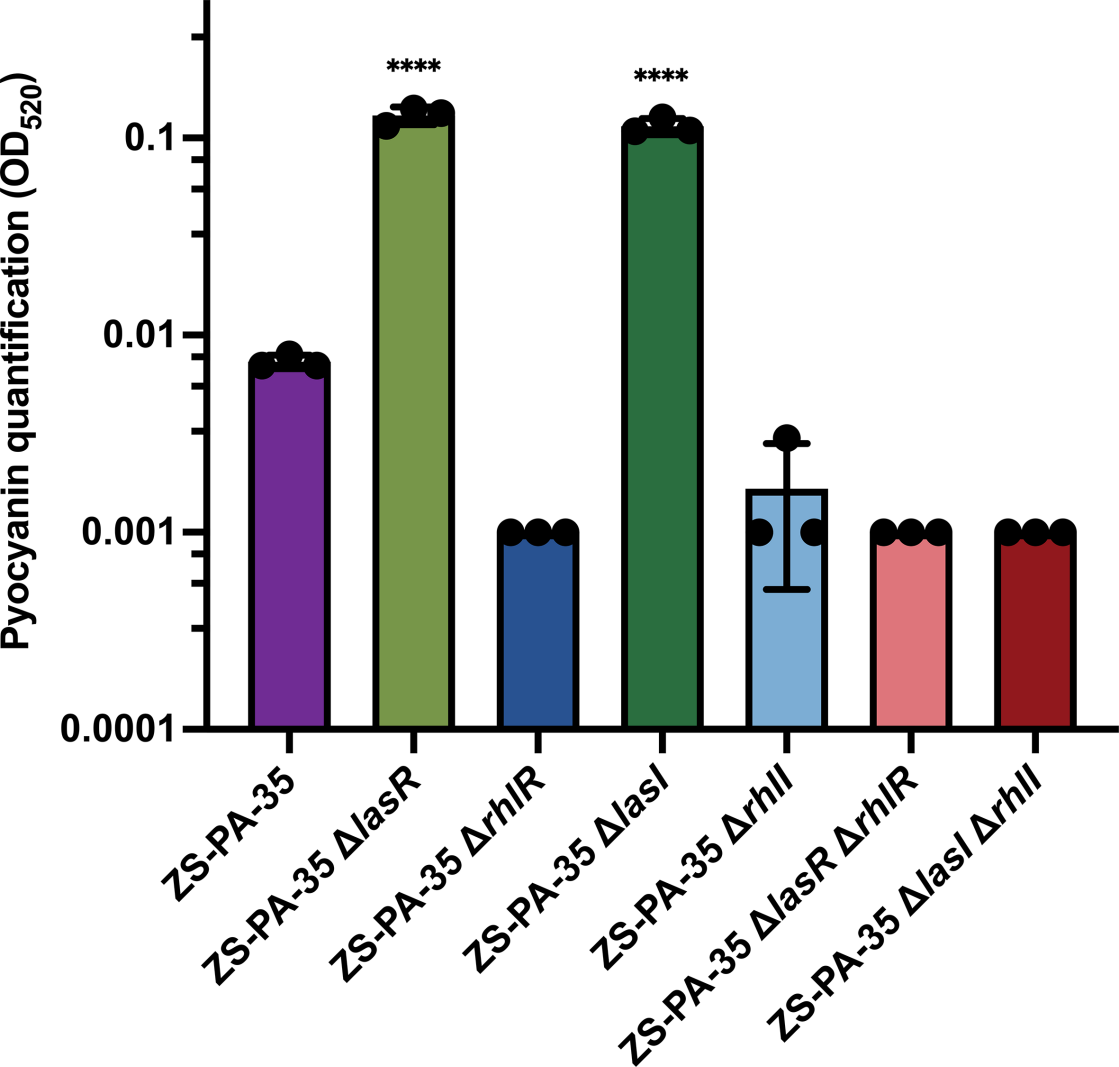
Quantification of pyocyanin production in LB medium for ZS-PA-35 and its QS mutants (Δ*lasR*, Δ*rhlR*, Δ*lasI*, Δ*rhlI*, Δ*lasR* Δ*rhlR*, and Δ*lasI* Δ*rhlI*, ZS-PA-35 Δ*lasR* Δ*rhlR*, and ZS-PA-35 Δ*lasI* Δ*rhlI*) at an OD_600_ of 1.0. Pyocyanin levels were measured by absorbance at OD_520_ using 1.5 mL cuvettes. Data are represented as mean ± SD. ****P < 0.0001 as determined by one-way analysis of variance with Dunnett’s multiple comparison test.

To explore the regulatory mechanisms of QS and contributions of the Las and Rhl systems in strain ZS-PA-35, six QS mutants were generated, including Δ*lasR*, Δ*rhlR*, Δ*lasI*, Δ*rhlI*, Δ*lasR* Δ*rhlR*, and Δ*lasI* Δ*rhlI*. To investigate the correlation between AI levels and various constructs, we measured the relative concentrations of 3OC12-HSL and C4-HSL (Table S1). Deletion of the Las system (Δ*lasR*, Δ*lasI*, Δ*lasR* Δ*rhlR*, and Δ*lasI* Δ*rhlI*) impacted the biosynthesis of both AHLs, whereas deletion of the Rhl system did not alter the level of 3OC12-HSL. Regarding C4-HSL, the deletion of the *rhlR* gene resulted in a twofold increase in its concentration compared to the wild-type strain (Table S1). This observation led us to hypothesize that the absence of its receptor may enhance its accumulation in cell-free supernatant (CFS), thereby driving the observed increase in C4-HSL levels. Subsequently, we measured pyocyanin levels in the cell-free lysogeny broth (LB) liquid to confirm the functionality of the QS mutants at an OD_600_ of 1.0, given that pyocyanin production relies on C4-HSL activation of RhlR. As anticipated, the absence of the Las system (*P* < 0.0001) resulted in a notable rise in pyocyanin production, whereas the deletion of the Rhl system modestly inhibited its production (Fig. 1). Taken together, the regulation of *lasR* and *rhlR* mRNA levels, along with phenotypic observations from QS mutants, suggests that the QS receptors in strain ZS-PA-35 are positively regulated in response to AI concentrations and cell densities.

### CFS enhances phage phipa2-induced bacterial cell lysis

**Fig 2:**
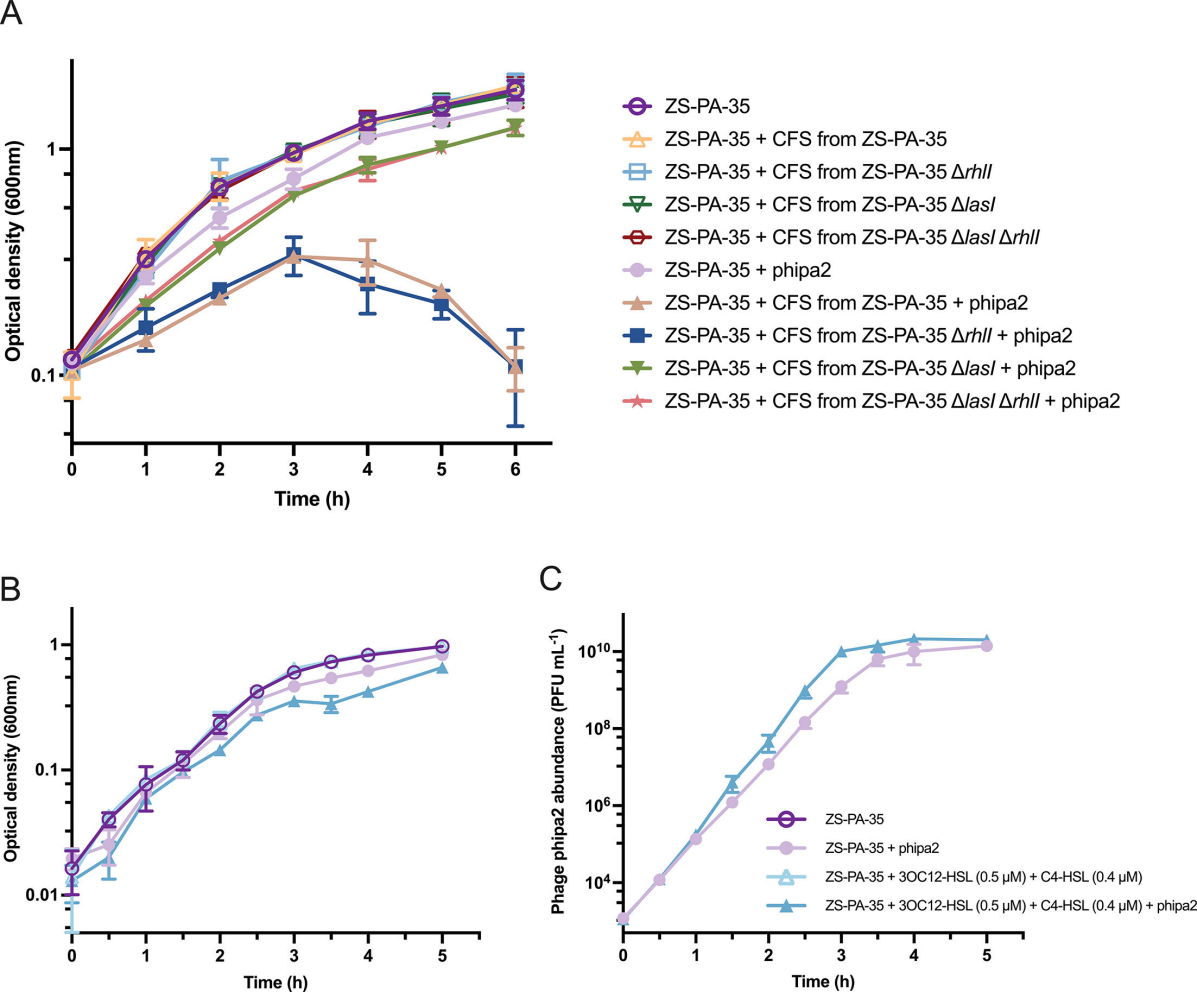
The effects of cell-free supernatant and synthetic AIs on phage-host interactions. (A) The growth dynamics of *P. aeruginosa* ZS-PA-35 were assessed in the presence or absence of phage phiPA2 at an MOI of 2, with measurements recorded hourly over a 6-hour incubation period. The experiments were conducted in conditioned medium obtained from high-cell-density (HCD) cultures of ZS-PA-35, ZS-PA-35 Δ*lasI*, ZS-PA-35 Δ*rhlI*, or ZS-PA-35 Δ*lasI* Δ*rhlI*, supplemented with 25% 4× LB medium. (B) Optical densities (OD_600_) of strain ZS-PA-35 were measured at an MOI of 0.01, with or without synthetic AI mixtures (0.5 µM 3OC12-HSL + 0.4 µM C4-HSL) in the presence or absence of phage phipa2 with an MOI of 0.01. (C) The corresponding phage concentration in the presence of synthetic AHL was quantified using a plaque assay. Data are averages of three samples with standard deviations (error bars). Statistical significance was determined by two-way analysis of variance with Dunnett’s multiple comparison test.

We next investigated the impact of CFS from HCD cultures of the WT strain, the double-synthase mutant (Δ*rhlI* Δ*lasI*), and individual synthase mutants (Δ*rhlI* and Δ*lasI*) of *P. aeruginosa* ZS-PA-35 on freshly inoculated WT cultures, both in the presence and absence of phage phipa2 at a multiplicity of infection (MOI) of 2. Notably, while the growth of ZS-PA-35 was unaffected by CFS alone, the addition of CFS from the WT strain (*P* < 0.0001) or ZS-PA-35 Δ*rhlI* (*P* < 0.0001) significantly increased the strain’s susceptibility to phipa2. This heightened sensitivity was evidenced by a dramatic and sustained reduction in optical densities within 6 h of incubation, ultimately leading to an optical density nearly 15-fold lower than that observed in LB medium by the end of the assay. In contrast, the CFS from the Δ*lasI* Δ*rhlI* double-synthase mutant (*P* < 0.0001) and the Δ*lasI* synthase mutant (*P* < 0.0001) exhibited a more moderate effect, comparable to that observed in LB medium (Fig. 2A). These findings underscore the intriguing role of extracellular factors, especially 3OC12-HSL produced by ZS-PA-35, in modulating phipa2 infection dynamics by accelerating its progression.

To further explore the role of synthetic AIs during phage infection, we assessed both bacterial density and phage production in the ZS-PA-35 strain with and without the addition of AIs with an MOI of 0.01. As a control, synthetic AI mixture and individual AIs were introduced into bacterial cultures, showing no impact on bacterial growth (Fig. 2B; Fig. S2A). Interestingly, the AIs induced slightly accelerated cell lysis by phipa2 compared to the control without AIs (*P* < 0.0001) (Fig. 2B). Moreover, the AIs showed minimal effect on accelerating the production of phipa2 (*P* < 0.0001), eventually reaching a concentration similar to the control without AIs, stabilizing at approximately ~1.7 × 10^10^ PFU/mL after 5 h (Fig. 2C). Unexpectedly, individual AI did not influence either phage susceptibility or production (Fig. S2A and B). These findings suggest that the ZS-PA-35 strain may exhibit a unique response to the concentrations of AIs used in this study, potentially due to variability in QS receptor sensitivity, as some strains are known to display hyper- or hypo-sensitive responses (25). Also, it is plausible that other unidentified AIs might regulate LasR and RhlR independently of LasI and RhlI synthases, contributing to the nuanced dynamics observed (26).

### *las*-mutants affect phage-host interactions in liquid medium

**Fig 3:**
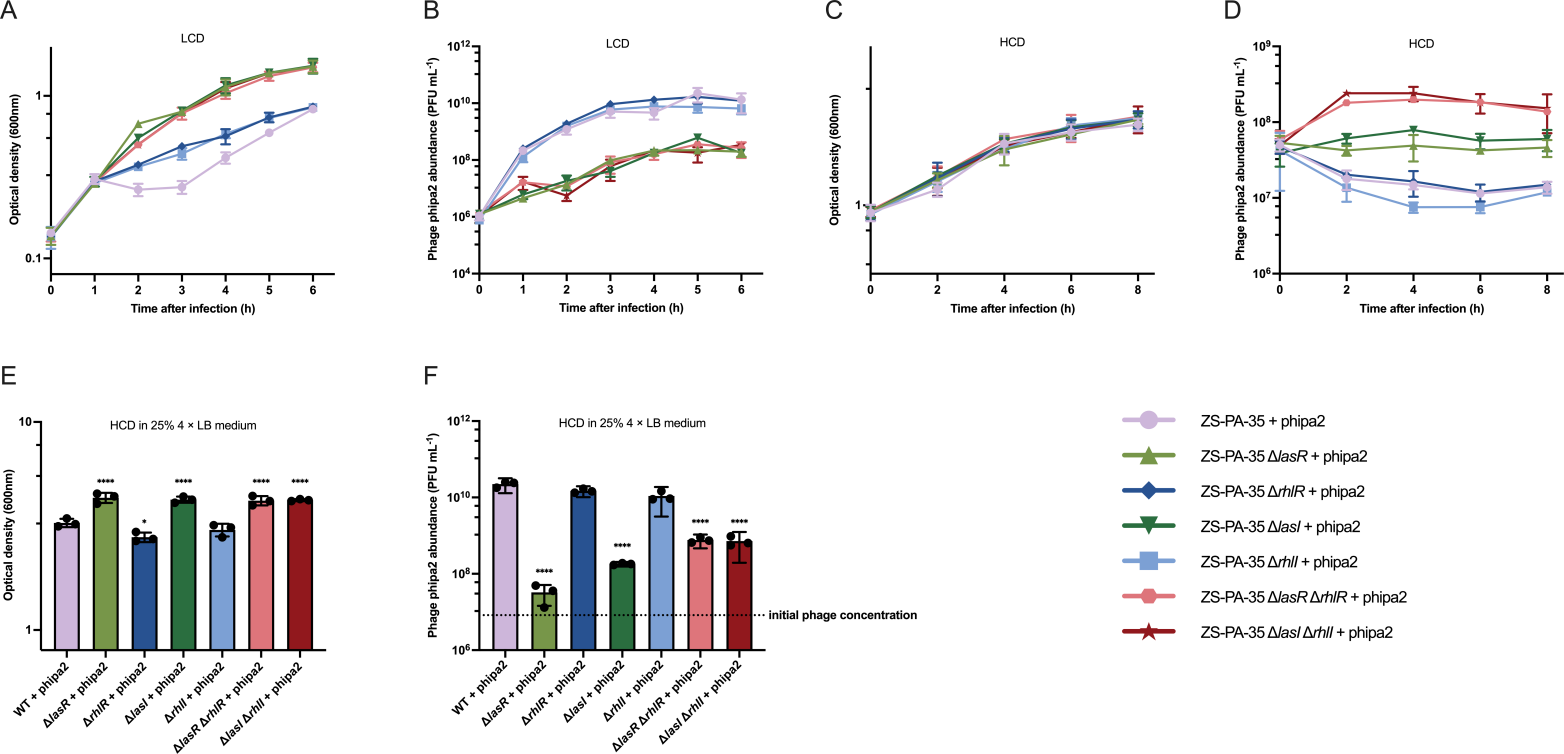
QS regulates phage-host interactions in ZS-PA-35. In LCD cultures, (A) OD_600_ and (B) corresponding phage concentrations of ZS-PA-35 and its QS mutants were determined at 1 h intervals over a 6 h period with phage phipa2 at an MOI of 0.01. In HCD cultures, similar measurements were taken at 2 h intervals over an 8 h period (C and D). The effect of nutrient depletion was analyzed by measuring optical density (E) and phage production (F) after supplementing HCD cultures with 25% 4× LB medium for 8 h. The dashed line indicates the initial concentration of phage phipa2. Statistical analysis was performed using two-way analysis of variance (ANOVA) (A, B, C, and D) or ordinary one-way ANOVA (E and F) with Dunnett’s test. Significance levels are indicated as *P < 0.05 and ****P < 0.0001.

To further investigate QS-mediated regulation of phage-host interactions, we evaluated the efficiency of plating (EOP) of phage phipa2 at an OD_600_ of 1.0. Our results revealed no significant differences in EOP among the QS mutants tested compared to their parental host strain (Fig. S3). As expected, when grown in LB medium, neither the single nor double deletion of QS genes had any visible impact on bacterial growth, with all strains displaying typical growth curves (Fig. S4). In the LCD cultures, all QS mutants exhibited reduced susceptibility to phage phipa2 at an MOI of approximately 0.01 following a 2 h incubation. The distinction between *las*- and *rhl*-mutants was evident in both optical density and phage concentration. As illustrated in Fig. 3A and B, both single (Δ*lasR* and Δ*lasI*) or double (Δ*lasR* Δ*rhlR* and Δ*lasI* Δ*rhlI*) mutants demonstrated greater resistance to phage infection compared to the Δ*rhlI*, Δ*rhlR,* and the wild-type, resulting in diminished phage phipa2 production (*P* < 0.0001). Despite the deletion of the Rhl system enhancing resistance to phipa2 infection compared to the wild-type, rapid phage production was observed in the Δ*rhlI*, Δ*rhlR* akin to that of the wild-type, where the phipa2 concentration stabilized at ~10^10^ PFU/mL. On the other hand, the phipa2 productions in the single (Δ*lasR* and Δ*lasI*) or double (Δ*lasR* Δ*rhlR* and Δ*lasI* Δ*rhlI*) mutants were approximately two orders of magnitude lower (*P* < 0.0001). Consistent with prior research, the Las system appears to play pivotal roles, holding a higher hierarchical rank than the Rhl system (16).

Yet, in HCD cultures, QS mutants exhibited distinct effects on phage-host interactions, particularly in terms of phage production at the same MOI as LCD. Notably, phage susceptibility remained consistent between the wild-type strain and QS mutants throughout the experiment (Fig. 3C). However, after 2 h, phage production increased by over twofold in the Δ*lasR* Δ*rhlR* and Δ*lasI* Δ*rhlI* cultures compared to the Δ*lasR* and Δ*lasI* cultures (*P* < 0.0001). Conversely, phage production decreased by more than threefold in the Δ*rhlR*, Δ*rhlI*, and wild-type cultures (Fig. 3D, *P* < 0.0001), indicating that the Las system may suppress phage proliferation under HCD conditions.

Next, we considered whether the minimal difference in phipa2 production among all the QS mutants could be due to its inability to replicate in cells at an early dormant stage, a condition potentially induced by the Las system. Indeed, QS has been reported to modulate and coordinate nutrient utilization and the homeostatic primary metabolism of individual cells. For example, a previous metabolic profiling study of *P. aeruginosa* revealed significant differences between a *lasI rhlI* double mutant and its wild-type strain. Identified metabolites showed alterations in the concentrations of tricarboxylic acid cycle intermediates, amino acids, and fatty acids (27). Likewise, in *Burkholderia glumae*, QS restricts glucose uptake and slows the primary metabolism of individual cells under crowded conditions (28). To investigate this, we introduced a 25% concentrated 4× LB medium into the HCD cultures premixed with phage phipa2, using an MOI of 0.01. This reactivation of the lytic cycle resulted in enhanced phage resistance in strains deficient in the Las system (Fig. 3E, *P* < 0.0001) and a more than 1,000-fold increase in phage progeny release after an 8 h incubation in Δ*rhlR*, Δ*rhlI*, and WT cultures (Fig. 3F, *P* < 0.0001). In contrast, phage production was less efficient in the Δ*lasR*, Δ*lasI*, Δ*lasR* Δ*rhlR*, and Δ*lasI* Δ*rhlI* cultures, with the strongest effect observed in the Δ*lasR* culture, where phage concentration increased by only fourfold relative to the initial phage input (Fig. 3F). This highlights that nutrient fluctuations during HCD conditions influence phage infection, ultimately leading to diverse outcomes in phage progeny production. In summary, our findings demonstrate that QS mutants exhibit distinct responses to phipa2, with the absence of the Las system conferring increased resistance during the early exponential phase. In contrast, dormancy induced by nutrient depletion and regulated by the Las system further suppresses phage proliferation, thereby protecting neighboring kin from infection. These findings underscore the bacterial capacity for flexible regulation of additional intracellular defense pathways, including those linked to metabolic status, with partial reliance on the QS system. By dynamically regulating the interaction between phage phipa2 and its hosts, bacterial populations can persist in an HCD state, ensuring lineage survival and minimizing the accumulation of mutations under phage predation pressure.

### Effect of baicalein on phage-host interactions in strain ZS-PA-35

**Fig 4:**
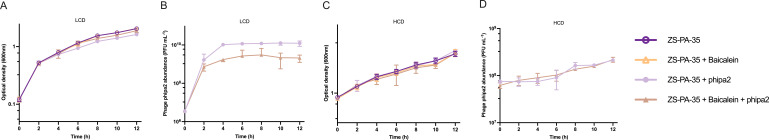
The impact of the QS inhibitor, baicalein, on phage-host interactions. Optical densities (LCD-A and HCD-C) and corresponding phage production (LCD-B and HCD-D) were recorded starting from both LCD (OD = 0.1) and HCD (OD = 1.0) cultures. Measurements were taken at 2 h intervals over a 12 h incubation period in LB medium, with or without the addition of 100 µM baicalein at an MOI of 0.01. Statistical significance was determined by two-way analsis of variance with Dunnett’s multiple comparison test. Error bars represent standard deviations from three replicates (*n* = 3).

Baicalein, recognized for its capacity to hinder QS-controlled phenotypes by binding to the QS transcriptional regulators, LasR and RhlR, and diminishing their ability to bind to the promoter region of target genes, may have implications for virulence repression and phage-host interactions (29, 30). To measure the effect of a QS inhibitor on phage-host infection dynamics, we exposed the wild-type ZS-PA-35 to phipa2 with an MOI of 0.01, with or without baicalein, at ODs of 0.1 and 1.0. In both ODs, the addition of baicalein to strain ZS-PA-35 did not impact bacterial growth at all (Fig. 4A and C). In agreement with results from the mutants lacking the Las system, at an OD of 0.1, baicalein significantly blocked phage proliferation, with a more than fivefold difference observed at 2 h intervals between 2 and 12 h, rendering the cells resistant to phipa2 infection compared to the control group without baicalein (Fig. 4A and B, *P* < 0.0001). However, at an OD of 1.0, there was no discernible difference in phipa2 susceptibility or production with or without baicalein, maintaining a steady concentration of phipa2 at approximately 10^8^ PFU/mL throughout the infection (Fig. 4C and D). The observed variations in inhibition levels at different MOIs, as shown in Fig. 2 to 4 for the control assays of phage phipa2’s inhibition of strain ZS-PA-35, likely stem from a combination of biological and technical factors. These factors include inconsistencies in bacterial and phage preparation, such as variations in culture conditions, growth phase synchronization, and phage titer accuracy, as well as environmental impacts like temperature fluctuations and differences in experimental handling. Despite these slight variations, the overall trends in the data consistently support the hypothesis that Las-mediated susceptibility to phage phipa2 is more pronounced during the early to mid-logarithmic growth phase, rather than when cells transition to HCD QS modes. These findings provide further evidence of the dynamic interplay between QS regulation and phage-host interactions.

### Phage adsorption and motility assays

**Fig 5:**
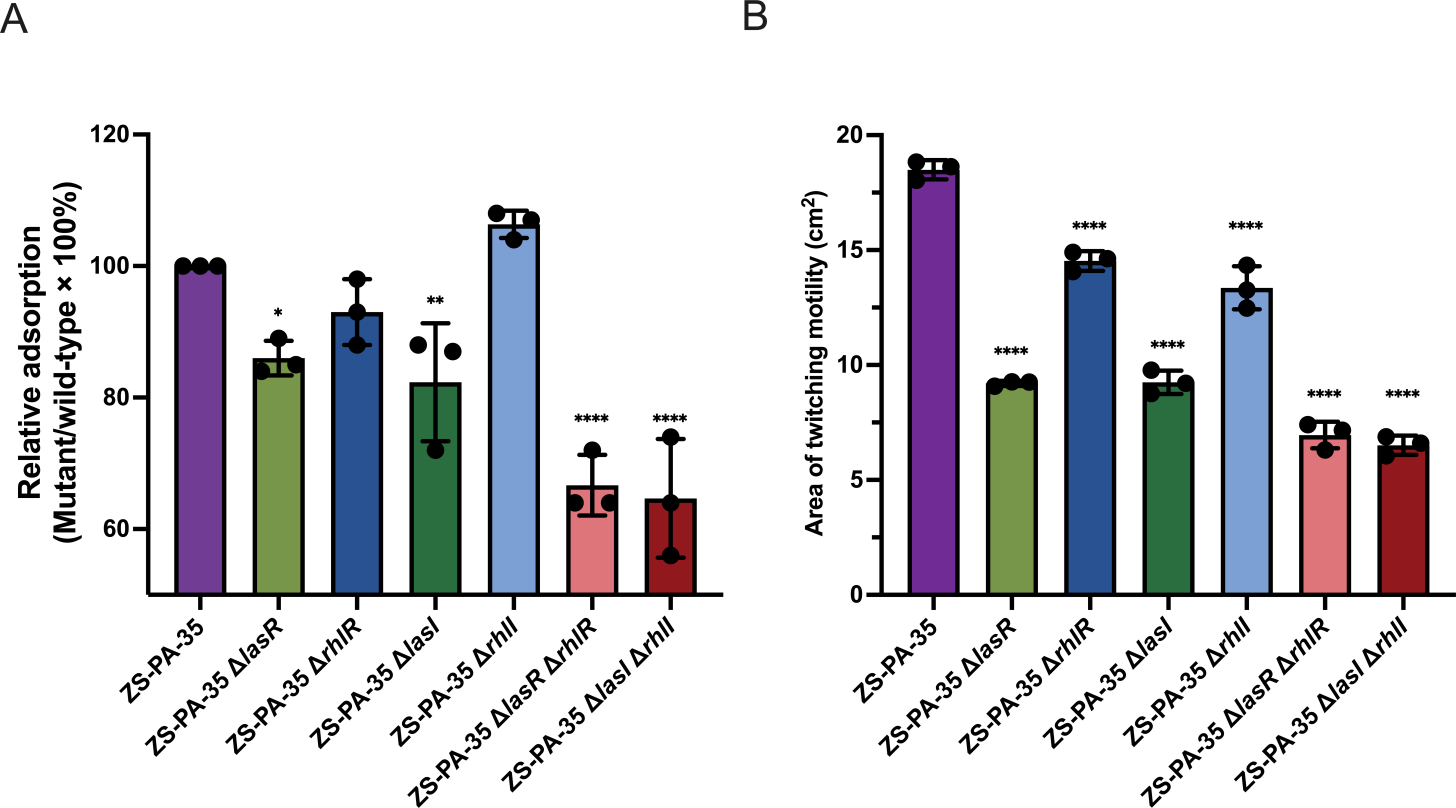
Phage adsorption and motility assay. (A) Adsorption assays were conducted to assess the interaction between phage phipa2 and its host strain ZS-PA-35, as well as QS mutants. (B) The impact of QS on twitching motility was evaluated by observing cell migration abilities at the agar-Petri dish interface. The data represent averages from three independent samples, with error bars indicating standard deviations. Statistical significance was determined using ordinary one-way analysis of variance with Dunnett’s test, with significance levels indicated as *P < 0.05, **P < 0.01, and ****P < 0.0001. Significant results are highlighted accordingly.

To address the question of how deletion of the Las system reduces the susceptibility of cells to phipa2, we measured phage adsorption efficiency by titrating unabsorbed phages after a 10 min incubation with the wild-type and QS mutants. As shown in Fig. 5A, phipa2 adsorbed less effectively to *las*-mutants (*P* < 0.05), with the double deletion mutants (Δ*lasR* Δ*rhlR* and Δ*lasI* Δ*rhlI*) having a more significant impact than the *las*-single deletion mutants (Δ*lasR* and Δ*lasI*). In agreement with the assays mentioned above, no significant differences were observed among the Δ*rhlR*, Δ*rhlI*, and wild-type ZS-PA-35 cultures (Fig. 5A).

*P. aeruginosa* utilizes T4P to facilitate a multicellular form of surface translocation known as twitching motility. Given that twitching motility indirectly reflects T4P functionality, we assessed the twitching motility of the wild-type strain and its QS mutants, providing insights into the interplay between QS and T4P dynamics. *P. aeruginosa* strains with a single QS gene deletion showed significant reductions in twitching motility compared to the wild-type. The impact of the Las system (Δ*lasI*, Δ*lasR*, Δ*lasR* Δ*rhlR*, and Δ*lasI* Δ*rhlI*) was even more noticeable, with an average decrease of ~56.95% in the distance traveled, compared to the Rhl system (*P* < 0.0001). As anticipated, mutations in the Rhl system (Δ*rhlI* and Δ*rhlR*) had a comparatively minor effect on motility, corresponding to only an ~24.61% decrease compared with the wild-type (Fig. 5B, *P* < 0.0001). Together, these results provide direct evidence that motility-related T4P is regulated by QS, as removing the Las system is associated with both decreased twitching motility and increased phage phipa2 resistance in strain ZS-PA-35. Interestingly, previous observations revealed that the accumulation of twitching-defective variants in the Δ*lasI* and Δ*rhlI* strains was primarily due to secondary mutations in the *cfr* and *algR* genes (31). These mutations underscore the intra-species diversity among *P. aeruginosa* isolates and highlight the potential for genetic adaptation under QS-disrupted conditions.

### The mutation in LasR abolishes QS regulation in strain ZS-PA-05

Non-synonymous mutations in LasR are highly prevalent among cystic fibrosis (CF) patients, often exhibiting social cheating behaviors, exploiting shared QS products without investing in their production costs. The consequences of these mutations for QS-mediated phage-host interactions remain largely unknown, adding unexpected complexity to QS regulation during clinical phage therapy. The EOP of phage phipa2 was approximately twofold higher in strain ZS-PA-35 than in strain ZS-PA-05 at an OD of 1.0 (Fig. S5). Paradoxically, the liquid medium proliferation consistently demonstrated lower phage yields in strain ZS-PA-35 compared to the clonal isolate strain ZS-PA-05. Additionally, bioinformatics analysis showed that strain ZS-PA-05 exhibited single nucleotide polymorphisms (SNPs) resulting in an amino acid substitution in LasR (L154P), while the rest of the QS genes remained identical to those of PAO1 at the amino acid level.

**Fig 6:**
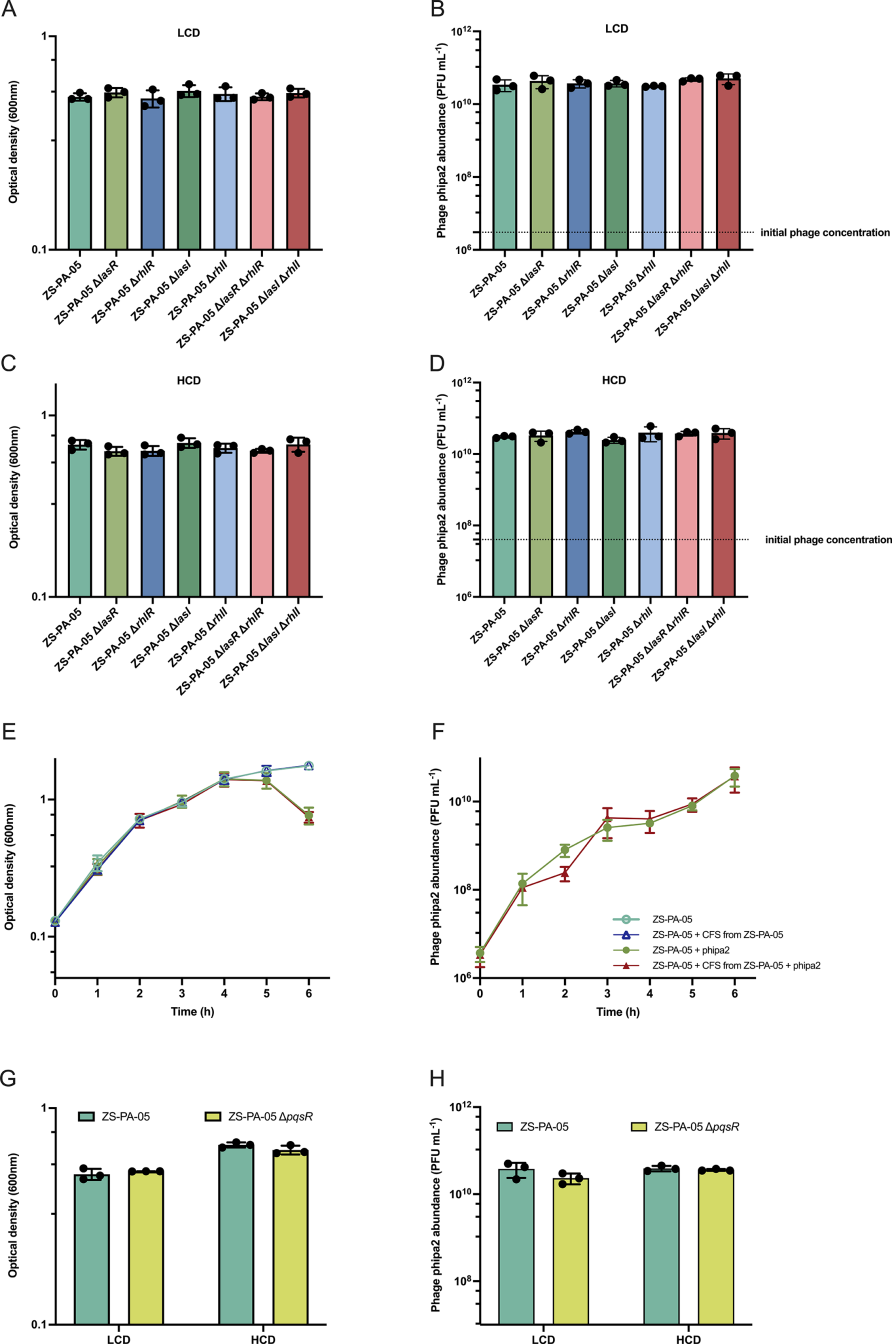
The mutation in LasR abolishes QS regulation of phage-host interactions in strain ZS-PA-05. (A, B, C, and D) Infection of ZS-PA-05 and its QS mutants with phipa2 under LCD and HCD QS modes. (A and C) Optical densities (OD_600_) and (B and D) phage phipa2 concentration were measured after an 8 h incubation period. The dashed line indicates the initial concentration of phage phipa2. CFS assay of interactions between ZS-PA-05 and phipa2 was analyzed by (E) optical densities and (F) phage production. (G) Optical densities (OD_600_) and (H) corresponding phage concentrations for ZS-PA-05 and ZS-PA-05 Δ*pqsR* were measured after an 8 h period with phipa2. All experiments were performed in triplicate at an MOI of 0.01. Data are represented as mean ± SD. The significance was analyzed by one-way analysis of variance (ANOVA) (A, B, C, and D) or two-way ANOVA (E, F, G, and H) with Dunnett’s multiple comparison test.

First, we quantified AIs in the CFS and found that, unlike strain ZS-PA-35, strain ZS-PA-05 exhibited significantly lower concentrations of AIs at an OD of 0.6 ~ 0.8, measuring in the 3.91 × 10^−3^ and 4.72 × 10^−5^ µM for both C4-HSL and 3OC12-HSL, respectively (Table S1). Theoretically, low AHL levels should suppress phage receptor expression at HCD, making the cell less susceptible to phage phipa2 infection. However, the observed increase in phage production by more than two orders of magnitude in strain ZS-PA-05 suggests that an amino acid substitution may completely eliminate LasR function (Fig. 6D). We then constructed corresponding QS mutants (Δ*lasR*, Δ*lasI*, Δ*rhlR*, Δ*rhlI*, Δ*lasR* Δ*rhlR*, and Δ*lasI* Δ*rhlI*) in strain ZS-PA-05 and measured both optical density (Fig. 6A and C) and phipa2 production (Fig. 6B and D) of incubation at initial ODs of 0.1 and 1.0, alongside with wild-type strain ZS-PA-05. Unlike strain ZS-PA-35, deletion of either the Las or Rhl system in strain ZS-PA-05 had no effect on phage proliferation, as phage titers stabilized at ~10^10^ PFU/mL measured by plaque assays with ZS-PA-35 acting as the hosts finally (Fig. 6B and D).

Additional signaling molecules in the CFS, such as PQS, could potentially reduce the production of pyocyanin and rhamnolipid, as well as susceptibility to phages (32). These concerns were ruled out by the CFS assay between strain ZS-PA-05 and phage phipa2, as well as deletion of gene *pqsR*, which demonstrated that the susceptibility of phipa2 to strain ZS-PA-05 was not dependent, if at all, on cell density, as neither phage susceptibility nor phage production was affected by the addition of CFS (Fig. 6E and F) or lacking gene *pqsR* (Fig. 6G and H). These results confirm previous studies indicating that *lasR* variants exist in clinical isolates, further supporting the notion that non-synonymous LasR mutations do not maintain protein activity in *P. aeruginosa* isolates colonizing intubated patients and may not respond to low autoinducer levels and fail to activate QS-regulated T4P-related phage-host interactions (33). It is important to acknowledge that genomic variations beyond the QS differences identified in this study may contribute to the phenotypic variations observed between strains ZS-PA-35 and ZS-PA-05. To investigate this, we compared the whole-genome sequences of ZS-PA-05 and ZS-PA-35, revealing a high degree of sequence identity and coverage (84% and 99.04%). Using DefenseFinder, we identified differential features in putative intracellular defense mechanisms, which may contribute to the observed variations in phage-host interactions (Fig. S6). This hypothesis is supported by a recent study demonstrating that QS in *Vibrio cholerae* enhances its cyclic oligonucleotide-based anti-phage signaling system, an abortive infection system, to mitigate phage predation (34). However, further investigation is needed to validate and fully elucidate these potential connections.

### Kin competition affects phage phipa2 proliferation

**Fig 7:**
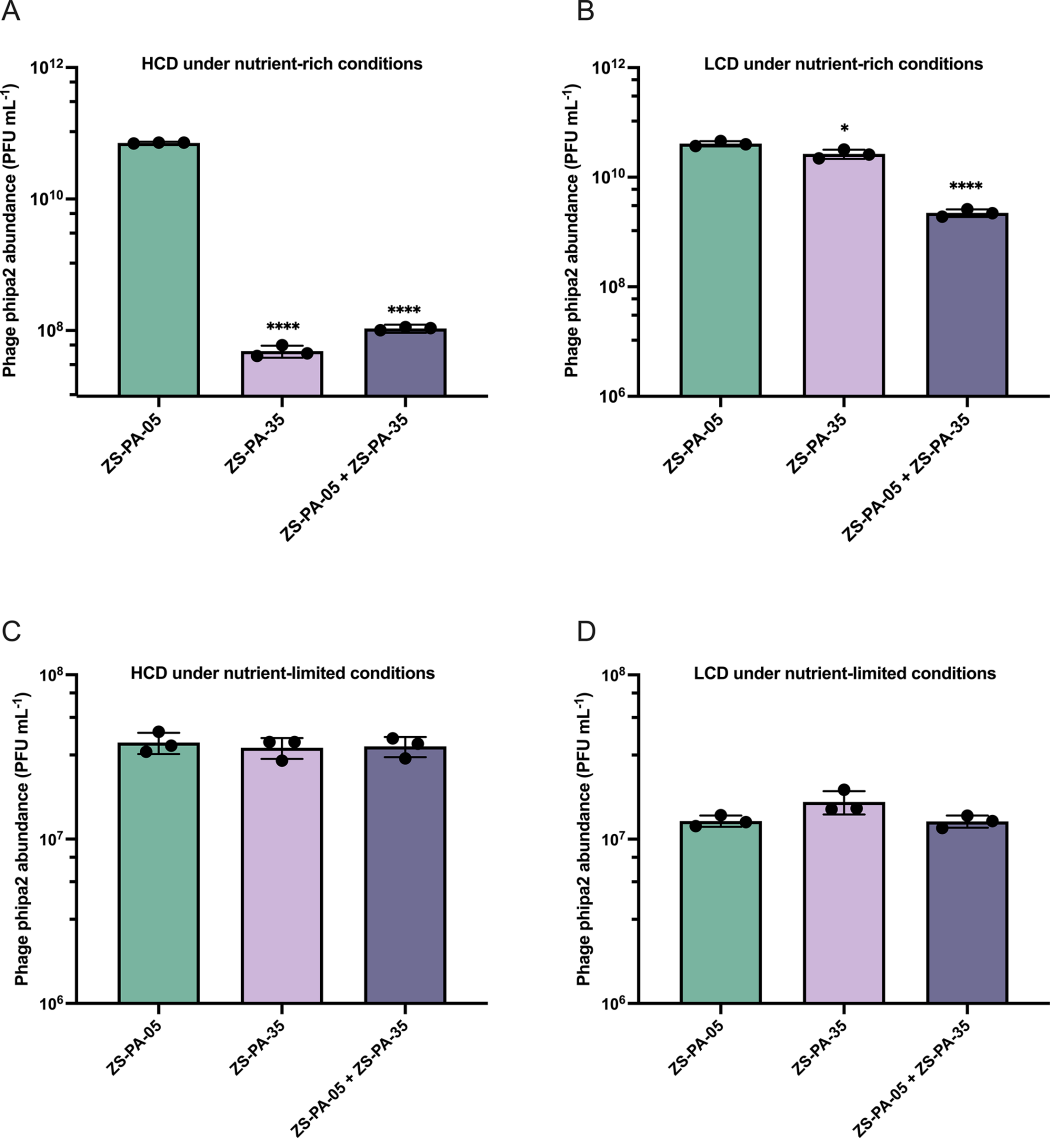
Concentrations of phage phipa2 during kin competition under (A and B) nutrient-rich and (C and D) nutrient-limited conditions. The concentration of phage phipa2 was measured from cultures of strains ZS-PA-05, ZS-PA-35, and a mixture of ZS-PA-05 and ZS-PA-35, all infected with phipa2 at both (B and D) LCD (OD_600_, 0.1) and (A and C) HCD (OD_600_, 1.0) over a (B and D) 6 h or (A and C) 8 h incubation period. Phage lysates were harvested, and phipa2 concentrations were enumerated using host strain ZS-PA-35. Statistical analysis was performed with one-way analysis of variance, with significance levels indicated as *P < 0.05 and ****P < 0.0001. Significant results are highlighted.

Loss-of-function mutations in *lasR* have been consistently observed during evolution experiments (35–37). These studies demonstrate that *lasR* mutants gain a competitive advantage by growth and optimizing resource utilization strategies. To simulate a heterogeneous infection environment, we co-cultured *P. aeruginosa* strains ZS-PA-05 and ZS-PA-35, followed by the addition of phage phipa2. This setup was designed to investigate potential synergistic interactions between the strains under LCD and HCD conditions in two media types representing nutrient-limited and nutrient-rich environments. In LB medium, strain ZS-PA-05, with altered QS, was able to support phage phipa2 proliferation at HCD. In contrast, strain ZS-PA-35, with a functional QS system, ceased phage proliferation, despite increased adsorption observed at HCD above. Indeed, phage concentration only reached ~10^8^ PFU/mL after 8 h in the mixed cultures, a 70-fold decrease compared to ZS-PA-05 (*P* < 0.0001), but a more than twofold increase compared to ZS-PA-35 in monocultures (Fig. 7A). Unexpectedly, under LCD conditions, we observed a significant drop in phage production when strains ZS-PA-35 and ZS-PA-05 were mixed with phage phipa2 (Fig. 7B, *P* < 0.0001). On the other hand, both strains individually supported phage production in the controls, showing more than a 100-fold increase, with levels ranging from ~10^7^ to 10^10^ PFU/mL (Fig. 7B). These results may suggest that phage phipa2 produced by “cheater” cells of strain ZS-PA-05, which have a loss of function in LasR, can be eliminated by quorum-competent “cooperator” cells of strain ZS-PA-35, irrespective of cell densities. From an evolutionary standpoint, we propose that strains with an altered QS system may gain an advantage by relying on signal producers to fully activate LasR in mixed environments. This strategy allows QS cheaters to reap the benefits of QS activation without bearing the metabolic cost of maintaining an active QS response. However, this advantage appears to diminish under low-nutrient conditions, as our observations revealed no significant difference in phage phipa2 production between monocultures and mixed cultures at either LCD or HCD inoculations (Fig. 7C and D). Using a cross-streak assay, we investigated the relative abundance of a bacterial mixture following phage phipa2 infection. Our results showed that the majority of the surviving and proliferating offspring originated from strain ZS-PA-35 under both nutrient conditions. Representative cross-streak images under nutrient-rich conditions are presented in Fig. S7. This highlights a critical challenge for phage therapy, as bacterial community heterogeneity can significantly complicate the dynamics of phage-host interactions. Variants in QS within the population may cooperate to reduce phage abundance, potentially undermining the effectiveness of phage therapy.

## DISCUSSION

*P. aeruginosa* has emerged as a critical public health threat, driven primarily by the increasing prevalence of antibiotic resistance, which complicates treatment and infection control strategies (1–3). Phage therapy offers a promising alternative; however, its efficacy is often hindered by the development of phage resistance (4–6). Recent discoveries reveal that *P. aeruginosa* strains PAO1 and PA14 employ QS to detect population density and fluctuations, thereby coordinating collective behaviors (16, 23). Notably, these emerging links between phage defense mechanisms and QS regulation highlight the intricate challenges of developing phage-based strategies to combat *P. aeruginosa* infections. However, the upregulation of phage receptor expression at HCD presents a fascinating paradox, as it could enhance the host’s susceptibility to phage adsorption, potentially influencing the dynamics of the long-term evolutionary arms race. Additionally, mutations in the QS master regulator LasR have been consistently observed in clinical isolates (20). The extent to which these non-synonymous mutations affect Las-mediated phage-host interactions remains to be explored.

In this study, we systematically explore the role of QS in regulating phage-host interactions in *P. aeruginosa* strains with both functional and altered QS systems. Our findings provide deeper insights into the adaptive strategies employed by the clinical isolate studied, revealing that they utilize a finely coordinated network of complementary defense mechanisms. First, we show that the addition of CFS significantly enhances the susceptibility of *P. aeruginosa* strain ZS-PA-35 to phage phipa2. This enhancement was absent when CFS was derived from either the Δ*lasI* Δ*rhlI* double-synthase mutant or the Δ*lasI* mutant, providing direct evidence that the Las system mediates phage-host interactions and underscoring the hierarchical organization of the *P. aeruginosa* QS network, with the Las system positioned at the apex, orchestrating the expression of both the Rhl and PQS systems and regulating key virulence traits (38, 39). The phenotypic traits of the Δ*lasI* Δ*rhlI* and Δ*lasR* Δ*rhlR* double mutants closely resemble those of the Δ*lasI* and Δ*lasR* single mutants, highlighting the dominant role of Las system disruption in suppressing phage phipa2 production under LCD conditions. These observations are consistent with prior studies, which reported that deletion of *rhlI* had no effect on plaque morphology, whereas deletion of *lasI* was linked to enhanced phage resistance (40).

**Fig 8:**
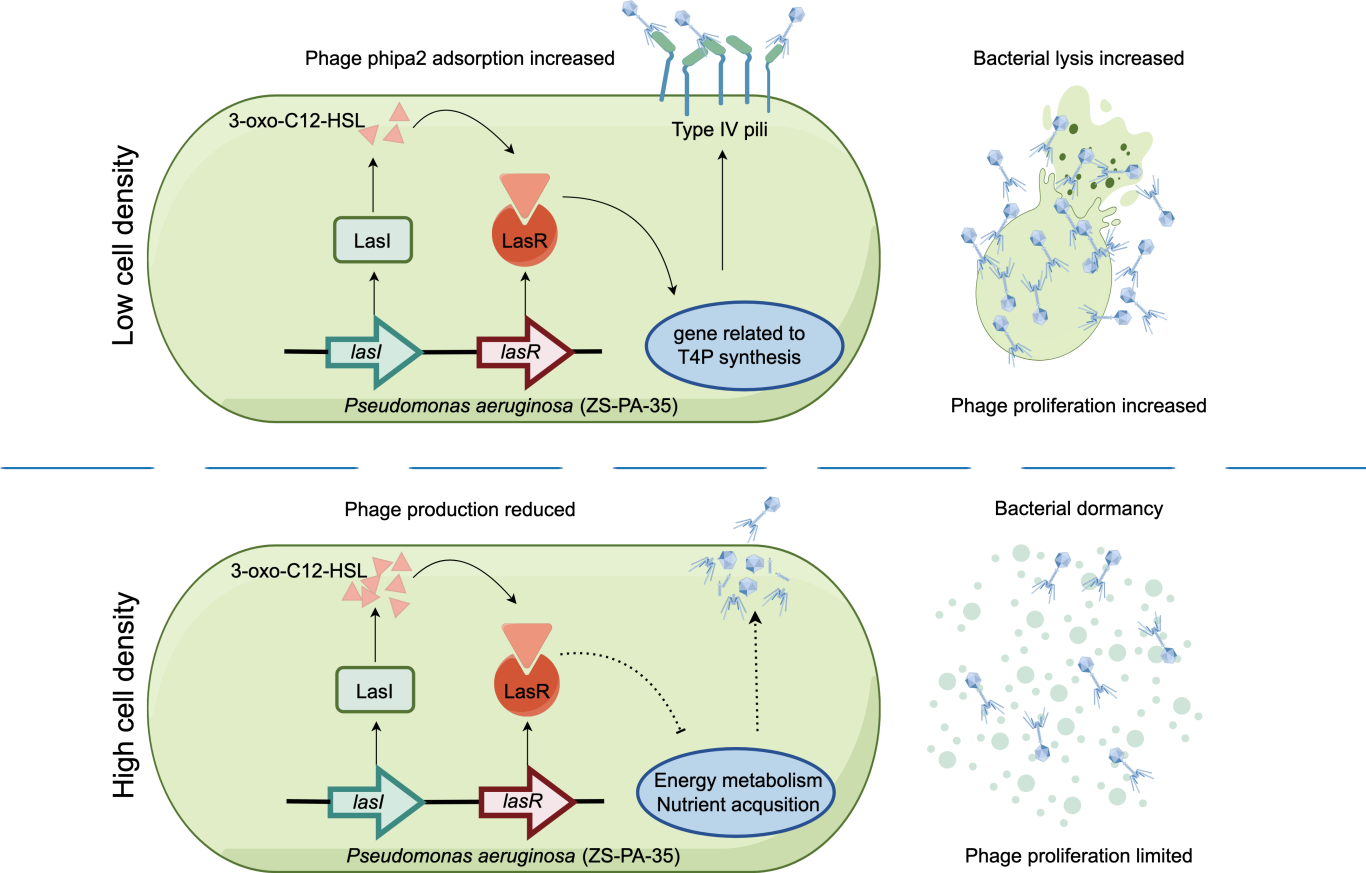
Schematic illustration of how the Las system regulates interactions between *P. aeruginosa* ZS-PA-35 and the T4P-dependent phage phipa2. The Las system modulates cell susceptibility to phage phipa2 and promotes bacterial lysis during the early exponential phase. Conversely, Las-regulated dormancy, triggered by nutrient depletion, suppresses phage proliferation, thereby protecting neighboring kin from infection under high-density conditions.

Intriguingly, Las-mediated phage phipa2 inhibition diminishes at HCD, as reflected by slightly increased phage production in double mutants lacking both the Las and Rhl systems compared to single mutants or the wild-type ZS-PA-35. We speculate that the contrasting dynamics observed between strain ZS-PA-35 and phipa2 at LCD and HCD may be attributed to dormancy induced by nutrient depletion, a process upregulated by the Las system. Indeed, QS-regulated global metabolic states have been shown to influence key physiological changes, including reduced membrane fluidity and enhanced chemical stability (27). Similarly, recent work on *V. campbellii* DS40M4 revealed that under glucose-depleted conditions, the wild-type strain outcompetes its QS-defective mutant counterparts (41). A plausible explanation is that cells utilize a functional QS system to suppress their metabolic activity, thereby maintaining a dominant population and minimizing the emergence of QS-deficient “cheater” subpopulations (42). Thus, we propose that phipa2 initially attaches to nascent dormant cells of the strain ZS-PA-35, entering a quiescent state synchronized with the host’s low-energy physiology. Upon improved nutrient availability, the phage likely transitions to an active lytic cycle, leveraging the host’s metabolic resurgence for replication and dissemination, as illustrated in Fig. 8. In line with this, a recent observation revealed that phage T1 relies on both AI-2 and metabolic cues mediated by cyclic AMP to initiate its lytic cycle in *Escherichia coli* ATCC 15144 (43). From an evolutionary perspective, bacteria benefit from utilizing QS to orchestrate key defense strategies, such as regulating phage receptor expression and inhibiting phage progeny production. This dual mechanism provides bacteria with remarkable flexibility, facilitating long-term coexistence with phages in dynamic environments. Furthermore, it prevents the emergence of QS-deficient mutants in mixed cultures, underscoring the critical role of QS in maintaining population stability under nutrient-limited conditions (41). Together, these findings highlight the complex interplay between mutational and non-mutational defense mechanisms prevalent in microbial communities.

However, the addition of synthetic AIs only subtly influenced bacterial susceptibility, contrasting with previous findings where exogenous 3OC12-HSL fully restored phage vB_Pae_QDWS susceptibility in both Δ*lasI* and Δ*lasI* Δ*rhlI* mutants of strain PAO1. Likewise, the introduction of AIs was observed to increase the expression of the *cas* gene to the level seen in the wild-type strain PA14 (15). We propose that this discrepancy underscores potential strain- or phage-specific variations in QS-mediated regulatory mechanisms. For instance, factors such as protein stability and the presence of HSL-degrading enzymes likely contribute to the complexity of these regulatory interactions (16, 44, 45). Beyond strain-specific responses to AIs, the subtle differences observed between LB medium and the CFS from Δ*lasI* Δ*rhlI* mutants point to the possibility that a broader spectrum of environmental and metabolic cues converge to fine-tune phage-host interactions. For instance, in addition to the canonical homoserine lactone AIs, the CFS from the *rhlI* mutant contains signaling molecules capable of stimulating gene expression regulated by the Rhl system (26). Similarly, in *V. cholerae*, bacterial biofilm formation has been shown to rely on elevated cytosolic AI-2 levels and the polyamine norspermidine, despite these molecules exerting opposing effects on biofilm formation when acting independently. Notably, exceptions to QS-regulated twitching motility have been documented within *P. aeruginosa*. For instance, in PAO1, a *lasIR rhlIR* quadruple knockout exhibited reduced levels of protease and elastase, undetectable levels of QS signal molecules (3OC12-HSL and C4-HSL), and minimal pyoverdine production, yet retained full twitching motility (46). Moreover, a previous study conducted a systematic examination of the Las and Rhl systems across 46 distinct growth conditions and revealed that the relative timing and intensity of expression for these two systems varied substantially depending on nutrient availability, and even their activity displayed no consistent correlation with absolute cell density (47). Collectively, these studies highlight the intricate and dynamic regulatory networks that bacteria utilize to adapt and thrive in complex, fluctuating environments, showcasing their remarkable resilience and versatility. From an applied perspective, uncovering this molecule could offer a promising avenue for the development of novel antimicrobials in synergy with phage therapy.

Baicalein, a bioactive flavonoid, exhibits a wide range of pharmacological activities, including antimicrobial and anti-inflammatory effects (48). Recent studies have demonstrated that the addition of baicalein to *P. aeruginosa* cultures disrupts biofilm formation, suggesting a potential role in modulating bacterial communication (49). Mechanistic investigations have further revealed that baicalein interacts directly with QS receptors, impairing their ability to bind DNA at QS-regulated promoters (30). In the experiments conducted here, the QS inhibitor baicalein selectively modulated phage susceptibility and progeny production during the early mid-logarithmic growth phase. In contrast, the addition of this compound to HCD cultures had no detectable effect on phage-host interactions, indicating a phase-dependent modulation of bacterial susceptibility to phages by QS inhibition. Our findings indicate that targeting QS through antagonism of autoinducer-binding receptors may be particularly effective during the LCD QS mode. The mechanisms underlying baicalein’s differential effects merit further investigation, especially to determine whether destabilized LasR retains its ability to bind promoter regions of target genes. Importantly, our results suggest that combining phage therapy with a QS inhibitor may not necessarily enhance its efficacy against this clinically significant pathogen, contrary to prior suggestions. This is primarily because QS inhibitors influence phage susceptibility and production during LCD but have no effect on T4P-mediated phage lysis or production at HCD conditions. Additionally, a notable concern arises from the *P. aeruginosa* PA14 model, where QS inhibition was shown to reduce the adsorption rate of the DMS3vir phage to its host. This reduction is linked to the inhibition of T4P. As a result, the destruction of bacterial cultures is delayed, which promotes the development of CRISPR immunity (50). Therefore, blocking QS could potentially lower, not enhance, the effectiveness of treatments that target the pilus-specific phage.

In natural environments, bacterial species and phages coexist in dynamic interplay, with cellular density fluctuating in response to nutrient availability (51). Our findings reveal that maintaining cells in an LCD state significantly enhances the efficiency of lysis, perhaps facilitating the development of phage resistance and promoting individual bacterial survival. In contrast, populations in an HCD state are more vulnerable to phage predation and nutrient depletion. This heightened risk stems from the upregulation of phage receptors driven by QS, coupled with the early transition of individual cells into dormant states. However, mutating receptors can be costly, potentially due to their essential roles in pathogenicity and other microbial phenotypic traits, as well as the allocation of resources that could otherwise be utilized for growth. This is particularly true in phipa2 mutants, as prior genomic comparisons indicate that phipa2 infection leads to the emergence of mutants including hyperpiliated *pilT* mutants. Thus, from the perspective of the microbial community with competition from kin cells, it is plausible that the production of QS signaling molecules by *P. aeruginosa* strains or the neighboring bacterial community could activate QS-mediated phage receptor expression, blocking phage proliferation, lowering phage concentration, and thereby protecting susceptible cells from extermination. This hypothesis was further supported by an expanded investigation that included strains with altered QS systems, enabling a more comprehensive understanding of their biological implications. Our findings corroborate previous reports that LasR variants commonly exhibit a loss of QS functionality (52).

However, our observation challenges the prevailing hypothesis that such mutations confer a fitness advantage over QS-functional isolates in the context of phage-host interactions. In our intraspecific competition assays conducted in rich media, the majority of LasR variants were outcompeted and ultimately eliminated under phage predation pressure. These findings underscore the nuanced and context-dependent role of QS in phage regulation within mixed microbial environments characterized by intense competition and predation. Such environments frequently expose cells to genetically diverse phage populations and neighboring kin. If nutrient depletion-dependent strategies are indeed widespread among *P. aeruginosa* isolates, strains with intact QS systems may activate collective responses to offset the fitness disadvantages associated with non-synonymous LasR mutations, as observed in strain ZS-PA-05. Notably, ZS-PA-05 is a multi-lysogen harboring several active prophages. By enabling the production of high quantities of phage phipa2 during HCD conditions, this strain may provide a substantial selective advantage to these prophages, facilitating their excision and release. These active prophages can subsequently infect non-lysogenic cells in the community, promoting horizontal gene transfer and enhancing the adaptability of the microbial population (Li et al., unpublished data).

The precise mechanisms underlying QS-mediated phage defense remain unclear. However, our findings underscore the critical need for a comprehensive understanding of phage-host interactions and their responses to environmental cues to optimize the efficacy of phage therapy against multidrug resistant (MDR) *P. aeruginosa* infections. Broadly, our study demonstrates that QS modulates diverse phage defense strategies, ranging from enhancing phage adsorption to suppressing phage production. These insights highlight the inherent complexity of phage-host dynamics, posing significant challenges for the design and implementation of clinical trials in phage therapy. Furthermore, whether the regulatory mechanisms identified here extend to other T4P-associated phages and clinical isolates remains an open question. Given that certain *P. aeruginosa* clinical isolates have evolved to bypass the QS master regulator to mitigate the regulatory burden, caution is warranted when extrapolating these findings. Such variability emphasizes the necessity of tailored approaches in translating these insights to clinical applications.

## MATERIALS AND METHODS

### Bacteria, phages, and growth conditions

Bacteria were cultured at 37°C in liquid LB (containing peptone 10 g, yeast extract 5 g, and NaCl 10 g/L) with agitation (220 rpm) and 1.5% LB agar plates. Phages phipa2 and phipa10 were proliferated using plaque assay and stored at 4°C until needed. A detailed characterization of phage phipa2 and phipa10 had been provided recently (7). All mutants developed in this study were derived from the wild-type strain of *P. aeruginosa* ZS-PA-35 and ZS-PA-05 (refer to Table S2). Antibiotics were used at the following concentrations: 30 µg/mL gentamicin and 100 µg/mL irgasan for *P. aeruginosa* and 15 µg/mL gentamicin for *E. coli*.

### Bacterial genome manipulation

The mutants were constructed through chromosome in-frame deletion of target genes based on allelic exchange (23). In brief, the regions upstream and downstream of the target sequence were amplified using PCR with primers that overlapped a common sequence. These amplified regions, which contained corresponding restriction sites, were used as templates to generate the sequence surrounding the gene of interest. The PCR product was then digested with the FastDigest Restriction Enzyme (Thermo Fisher Scientific, CA, USA) and subsequently cloned into the pEXG2 plasmid. This recombinant plasmid was introduced into the competent SM10 cell via heat shock and then conjugated into wild-type strains. The resulting transconjugants were successfully selected on LB agar plates supplemented with 30 µg/mL gentamicin and 100 µg/mL triclosan. To ensure plasmid loss, they were subsequently recovered by restreaking on low-nutrient 10% sucrose LB agar plates (containing peptone 5 g, yeast extract 1 g, NaCl 5 g, agar 15 g, and sucrose 100 g/L) and incubating at room temperature, enabling further refinement of the selection process. The desired mu.tants were confirmed by PCR and sequencing to check for the loss of the target gene. The primers used are listed in Table S3.

### Phage-host interaction in liquid medium

To prepare CFS, overnight cultures of strains ZS-PA-35, ZS-PA-35 Δ*lasI*, ZS-PA-35 Δ*rhlI*, ZS-PA-35 Δ*lasI* Δ*rhlI*, and ZS-PA-05 were diluted 1:1,000 into 15 mL fresh medium in 50 mL tubes and grown to an OD_600_ of 1.0. The supernatant was collected, sterilized through 0.22 µm filters (Millipore, MA, USA), and stored at −20°C for further analysis. Concentrated LB medium (4×) was added to the CFS at a final concentration of 1× to maintain nutrient consistency. Phages were introduced at a low bacterial density with an MOI approximately at 2. Samples were collected, and cell density was determined by measuring OD_600_ using a spectrophotometer (SP-UV 200, Shanghai Spectrum Instruments Co. Ltd.) with 1.5 mL Biofil cuvettes (CUV010015).

To evaluate the impact of QS gene deletion on bacterial growth, standard growth curves were generated by inoculating overnight cultures into 15 mL of fresh LB medium, starting from both LCD (OD_600_ = 0.002) and HCD (OD_600_ = 1.0), followed by an 8 h incubation. For the phage inhibition assay, overnight cultures were diluted to an initial OD_600_ of approximately 0.01. Phages were introduced to cultures at OD_600_ values of 0.1 and 1.0 to assess their inhibitory effects under different bacterial growth conditions, using an MOI of 0.01. Bacterial growth and phage concentration were monitored by measuring OD_600_ and performing plaque assays.

To assess the effects of nutrient availability on phage-host interactions, cultures were supplemented with 25% 4× LB medium and phipa2 at an MOI of 0.01 when ZS-PA-35 or its QS mutants reached an OD_600_ of 1.0 in LB medium. Optical densities and phage abundance were quantified after 8 h of incubation.

The effects of individual and combined AIs were evaluated by adding 3OC12-HSL (0.5 µM, Merck) and C4-HSL (0.4 µM, Merck) to LB medium, either separately or in combination, as previously established for strain PA14 (15). Phipa2 was added at an OD_600_ of 0.01 and an MOI of 0.01. To assess the effects of QS inhibitors, wild-type strains were cultured in LB medium with baicalein (100 µM, Merck), and phages were introduced once the optical density increased from 0.01 to 0.1. Bacterial density and phage concentration were monitored at 2 h intervals.

### Phage adsorption assay

Overnight cultures of ZS-PA-35 were diluted to an OD_600_ of 0.01 in fresh LB medium and incubated to the mid-log phase. Phages were introduced at an MOI of 0.001. After a 10 min incubation, the mixture was promptly centrifuged at 12,000 × *g* at 4°C for 3 min. The supernatant was then diluted in saline buffer containing 10 mM MgSO_4_ and 5 mM CaCl_2_, and a plaque assay was conducted to determine the quantity of unabsorbed phages. The relative adsorption was normalized to ZS-PA-35 for each assay.

### Autoinducer quantification assay

Quantification of the autoinducer produced by *P. aeruginosa* was conducted through ultra-high-performance liquid chromatography-high resolution mass spectrometry (UHPLC-HRMS) analysis. Sample preparation involved diluting overnight strains by 1,000-fold and culturing until reaching optical densities of 0.6 ~ 0.8. Cell-free supernatant was harvested, followed by mixing with ultrasonics and extraction with 10 mL of ethyl acetate at 4°C for 1 h. Centrifugation at 12,000 × *g* for 5 min yielded supernatant, subsequently dried with nitrogen, redissolved in 200 µL of methanol, and filtrated (0.22 µM, Millipore, MA, USA) for high performance liquid chromatography-mass spectrometry (HPLC-MS) analysis. The instrumental setup comprised a 1290 Infinity II LC System (Agilent, USA) and a QTRAP 6500 LC-MS/MS System (SCIEX, USA). A ZORBAX Eclipse Plus C18 column (4.6 × 30 mm, 3.5 µm particle size, Agilent) served as the stationary phase, with solvents A and B as mobile phases for analyte separation. Mobile phase A constituted an aqueous solution of 0.125% formic acid plus 2 mM ammonium acetate, while mobile phase B consisted of an acetonitrile solution of 0.125% formic acid plus 2 mM ammonium acetate. The flow rate was set at 300 µL/min, with the column temperature maintained at 30°C. The binary gradient initiated at 20% mobile phase B and escalated to 90% mobile phase B over 6 min. Following a 3 min hold at 90% mobile phase B, the composition reverted to 20% mobile phase B within 0.1 min and was held for an additional 7.9 min to equilibrate the column. Samples were injected in 2 µL volumes. An external calibration curve, complemented by quality control samples, was employed to ensure accurate quantification of analytes and to validate system stability. Additionally, *P. aeruginosa* type strain PAO1 was included as a reference. Data analysis was performed using MultiQuant 3.0 software (SCIEX, USA).

### Bacterial competition and cross-streak assays

To determine the proliferation capacity of phage phipa2 under heterogeneous conditions, bacterial competition assays were conducted at an MOI of 0.01 under both nutrient-rich and nutrient-limited conditions. LB medium is used to simulate nutritional adequacy. The nutrient-limited medium was composed of a modified M9 medium supplemented with 0.4% glucose, 10 mM MgSO_4_, and 10% LB. Under LCD conditions, equal aliquots of ZS-PA-05 and ZS-PA-35 were combined at an initial OD of 0.1 to create a bacterial mixture. Phage phipa2 was then introduced to monocultures of ZS-PA-05, ZS-PA-35, and the bacterial mixture. Optical densities and phage concentrations were measured after a 6 h incubation period. In HCD cultures, similar measurements were taken after an 8 h incubation at an initial OD of 1.0.

A cross-streak assay was conducted to evaluate the relative abundance of each strain in mixed cultures accordingly (53). Briefly, phage lysates were plated and purified on LB agar supplemented with 50 mM sodium citrate to destabilize phage particles, thereby facilitating colony formation. From the surviving colonies, 32 individual isolates were selected for further analysis. These isolates were subjected to a cross-streak assay using the phage phipa10, which selectively inhibits the growth of ZS-PA-35 but not ZS-PA-05. In this assay, phipa10 lysate was streaked across LB agar plates, followed by the perpendicular streaking of the bacterial isolates. ZS-PA-35 and ZS-PA-05 were included as controls on the same plate. The plates were incubated at 37°C for 24 h to allow for the differentiation between the two strains. All bacterial competition assays were performed in triplicate to ensure the reproducibility of the results.

### Twitching motility and pyocyanin assay

For twitching motility analysis, 2.5 µL of bacterial cultures with an OD_600_ of 0.1 were inoculated into the base of 20 mL of 1.5% LB agar plates. After a 3-day incubation period at 37°C, the agar was discarded, and the plate was stained with 5 mL of 0.4% crystal violet for 30 min. Any excess stain was gently rinsed off with tap water. The extent of twitching motility was quantified by measuring the area of the stain using ImageJ (https://imagej.net/ij/).

Pyocyanin was extracted and quantified following previous methods (54). In brief, overnight bacterial cultures were back-diluted to an OD_600_ of 0.001 and incubated until an OD_600_ of 1.0 was reached. CFS (5 mL) from the ZS-PA-35 wild-type strain and its QS mutants were harvested and combined with 3.5 mL of chloroform. The mixture was vortexed for 30 seconds and centrifuged at 10,000 × *g* for 5 min. The organic phases (3 mL) were collected and vortexed for 30 s with 1 mL of 0.2 M HCl, followed by a second round of centrifugation at 10,000 × *g* for 5 min. The pyocyanin in the pink aqueous phase was quantified by measuring the optical density at OD_520_ using 1.5 mL cuvettes.

### Genome comparison and visualizations

Analysis of the genomic alignment between ZS-PA-05 and ZS-PA-35 by blastn (https://blast.ncbi.nlm.nih.gov/Blast.cgi). The predicted phage defense systems were detected by DefenseFinder (https://defensefinder.mdmlab.fr/) (55). Links connecting colored circles represent similarities between ZS-PA-05 and ZS-PA-35, with only one best hit and using 85%–90% (yellow), 90%–95% (green), and >95% (blue) nucleic acid identity thresholds. Genomic regions were divided into 50 kb and counted for coding DNA sequence (CDS) density, GC content, and GC skew. The results were visualized by Circos v.0.69-8.

### Statistical analysis

Graphical depiction and statistical analysis were performed using GraphPad Prism 10.0.3 (GraphPad, La Jolla, CA) and Figdraw (https://www.figdraw.com/). Each experiment was independently repeated three times using distinct biological samples. The data were presented as mean ± standard deviation. Group differences were assessed using two-way analysis of variance (ANOVA) for Fig. 2, 3A through D, 4, 6E through H; Fig. S2 and S4, one-way ANOVA for Fig. 1, 3E and F, 5, 6A through D, 7; Fig. S3, or Student’s *t*-test for Fig. S5.

## Data Availability

The complete genome sequences of *P. aeruginosa* and phages are available in GenBank under the following accession numbers: ZS-PA-35 (GCA_020567355.1), ZS-PA-05 (SAMN25656732), phipa2 (OK539824.1) and phipa10 (OK539826.1). The data of the sources utilized to generate all main and supplemental figures in this study are accessible in the supplemental material.

## SUPPLEMENTAL MATERIAL

Supplemental file 1 aem.02402-24-s0001.pdf Tables S1 to S3; Figures S1 to S7.

Supplemental file 2 aem.02402-24-s0002.xlsx Data sources for statistical analyses.

ASM does not own the copyrights to Supplemental Material that may be linked to, or accessed through, an article. The authors have granted ASM a non-exclusive, world-wide license to publish the Supplemental Material files. Please contact the corresponding author directly for reuse.
