# Supplementary material for: Phage-host interaction in Pseudomonas aeruginosa clinical isolates with functional and altered quorum sensing systems
Source: Appl Environ Microbiol. 2025 Mar 4;91(4):e02402-24. doi: 10.1128/aem.02402-24 (PMC12016573; doi:10.1128/aem.02402-24)
Supplement: Supplemental file 1 — Tables S1 to S3; Figures S1 to S7. [file aem.02402-24-s0001.pdf]

Supplemenatry File 1. Table S1 to S4 and Figure S1 to S7

Table S1. Concentrations of autoinducers 3OC12-HSL and C4-HSL in *Pseudomonas aeruginosa* strains ZS-PA-35 and ZS-PA-05, along with their corresponding quorum sensing (QS) mutants. The PAO1 strain was included as a reference.

Strains	OD600	3OC12-HSL (μM)	C4-HSL (μM)
ZS-PA-35	0.642	0.327819	0.277695
ZS-PA-35 Δ*lasR*	0.652	0.003699	0.037209
ZS-PA-35 Δ*rhlR*	0.623	0.339790	0.577236
ZS-PA-35 Δ*lasI*	0.66	0.000538	0.041064
ZS-PA-35 Δ*rhlI*	0.693	0.340294	0.006133
ZS-PA-35 Δ*lasR* Δ*rhlR*	0.757	0.000773	0.015596
ZS-PA-35 Δ*lasI* Δ*rhlI*	0.699	0.000202	0.008470
ZS-PA-05	0.686	0.000047	0.003910
ZS-PA-05 Δ*lasR*	0.638	0.000074	0.001960
ZS-PA-05 Δ*rhlR*	0.668	0.000068	0.003430
ZS-PA-05 Δ*lasI*	0.699	No detection	0.001250
ZS-PA-05 Δ*rhlI*	0.676	0.000045	0.003590
ZS-PA-05 Δ*lasR* Δ*rhlR*	0.717	0.000104	0.003250
ZS-PA-05 Δ*lasI* Δ*rhlI*	0.706	No detection	0.001280
PAO1	0.627	0.698223	0.607294

Table S2. Bacterial strains, plasmids and bacteriophage

Name	Genotype or relevant markers
Escherichia coli	
SM10	λ-*pir*，*Escherichia coli*
Pseudomonas aeruginosa	
PAO1	Reference strain
ZS-PA-35	Wild-type, *Pseudomonas aeruginosa*, susceptible to phipa2 and phipa10
ZS-PA-05	Wild-type, *Pseudomonas aeruginosa*, susceptible to phipa2
ZS-PA-35 Δ*lasR*	In-frame deletion of *lasR* for ZS-PA-35
ZS-PA-35 Δ*rhlI*	In-frame deletion of *rhlI* for ZS-PA-35
ZS-PA-35 Δ*lasI*	In-frame deletion of *lasI* for ZS-PA-35
ZS-PA-35 Δ*rhlR*	In-frame deletion of *rhlR* for ZS-PA-35
ZS-PA-35 Δ*lasR* Δ*rhlR*	In-frame deletion of both *lasR* and *rhlR* for ZS-PA-35
ZS-PA-35 Δ*lasI* Δ*rhlI*	In-frame deletion of both *lasI* and *rhlI* for ZS-PA-35
ZS-PA-05 Δ*lasR*	In-frame deletion of *lasR* for ZS-PA-05
ZS-PA-05 Δ*rhlI*	In-frame deletion of *rhlI* for ZS-PA-05
ZS-PA-05 Δ*lasI*	In-frame deletion of *lasI* for ZS-PA-05
ZS-PA-05 Δ*rhlR*	In-frame deletion of *rhlR* for ZS-PA-05
ZS-PA-05 Δ*lasR* Δ*rhlR*	In-frame deletion of both *lasR* and *rhlR* for ZS-PA-05
ZS-PA-05 Δ*lasI* Δ*rhlI*	In-frame deletion of both *lasI* and *rhlI* for ZS-PA-05
ZS-PA-05 Δ*pqsR*	In-frame deletion of *pqsR* for ZS-PA-05
Plasmids	
pEXG2	Allelic exchange vector with pBR origin, Gm^R, *sacB*
pΔlasR	Gm^R; pEXG2 derivative deleting *lasR* in-frame of ZS-PA-35 and ZS-PA-05
pΔrhlI	Gm^R; pEXG2 derivative deleting *rhlI* in-frame of ZS-PA-35 and ZS-PA-05
pΔlasI	Gm^R; pEXG2 derivative deleting *lasI* in-frame of ZS-PA-35 and ZS-PA-05
pΔrhlR	Gm^R; pEXG2 derivative deleting *rhlR* in-frame of ZS-PA-35 and ZS-PA-05
pΔpqsR	Gm^R; pEXG2 derivative deleting *pqsR* in-frame of ZS-PA-05
Bacteriophages	
phipa2	*Podoviridae*, isolated from Zhongshan Hospital, Shanghai, China
phipa10	*Myoviridae*, isolated from Zhongshan Hospital, Shanghai, China

Table S3. Oligonucleotides used in this study

Name	Primers (5'-3')
lasR deletion	
lasR_1	TTTCTCGAGCACTTCCTCCAAATAGGAAGCTG
lasR_2	GTTTAAGAAGAACGTAGCGCTTTACTCTCTGATCTTGCCTCTCAGGTCGG
lasR_3	GATCAGAGAGTAAAGCGCTACGTTCTTCTTAAACTATTAACCAATCAGCC
lasR_4	TTTGGATCCCGGATCGCCCGGCCGAGAGC
lasR_5	GAGGTCACACCGAACTTCCG
lasR_6	GTTCTTCGAGGAAGCCTCGG
rhlI deletion	
rhlI_1	TTTTCTAGACCGTGGCGCGCGACCAGCAG
rhlI_2	GAGCGCGAAACGGCTGACGACCGACCAAGTCCCCGTGTCGTGCCGGCCGAG
rhlI_3	CACGGGGACTTGGTCGGTCGTCAGCCGTTTCGCGCTCTTTTTTCCGCTTCTC
rhlI_4	TTTGGATCCGCCTGGCGCTGGCGCTCCAG
rhlI_5	GGACGCTACCGGCATCAAG
rhlI_6	CCGTTGCGAACGAAATAGCG
lasI deletion	
lasI_1	TTTTCTAGAGGGCTGCGGTCTCGGCCTGC
lasI_2	CCTATTTGGAGGAAGTGAAGCGGGGACCTGTCGGCTCGCGCCGGCGCGTTCTC
lasI_3	GACAGGTCCCCGCTTCACTTCCTCCAAATAGGAAGCTGAAGAATTTATGC
lasI_4	TTTGGATCCCGTAGCGGCCATTATGGCCG
lasI_5	GATCTGGGTCTTGGCATTGAG
lasI_6	CGGTTGCTGGCGAATTCTCG
rhlR deletion	
rhlR_1	TTTTCTAGACCTACGCGCCACTGGGAGCC
rhlR_2	CCGGCGCGCCCTACGCTTGCAGTAAGCCCTGATCGATAAAATGCATCACAG
rhlR_3	CAGGGCTTACTGCAAGCGTAGGGCGCGCCGGCCGGCGCGCCCTACCAGATC
rhlR_4	TTTGGATCCGCGCGAAAGCTCCCATACCG
rhlR_5	CTATGGCGTGCGCCACAC
rhlR_6	GAACTCTTGCCGTCGGCG
pqsR deletion	
pqsR_1	TTTTCTAGATGACGATTGCAGGTTTCGG
pqsR_2	CTGCTGGAGAACGCTCCCTTATTCCTTTTATTGGGTGGCG
pqsR_3	TAAAAGGAATAAGGGAGCGTTCTCCAGCAGACGCTGGCCG
pqsR_4	TTTAAGCTTCCTCGATACCAGCATCGTC
pqsR_5	CAGCAACCTGGAAATCGA
pqsR_6	CGATGGTGATGGCGATAT

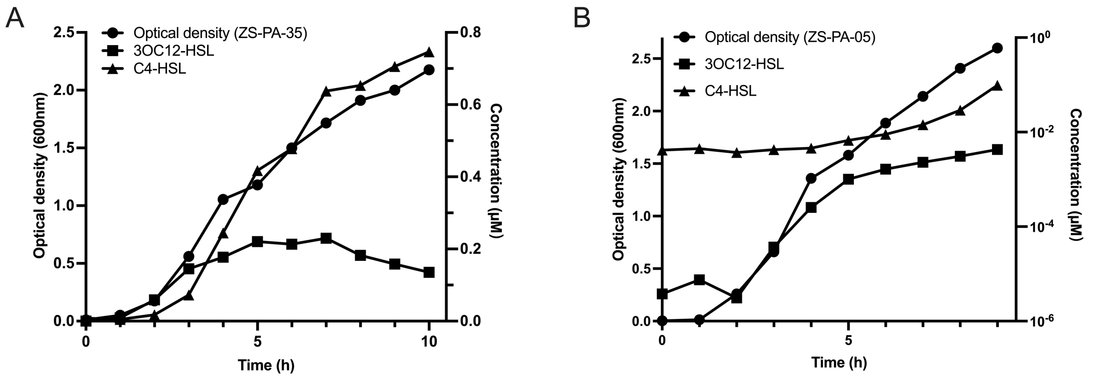

Figure S1. (A) Concentrations of AHL QS autoinducers (3OC12-HSL and C4-HSL) in strains ZS-PA-35 (A) and ZS-PA-05 (B) at various optical densities (OD_{600}).

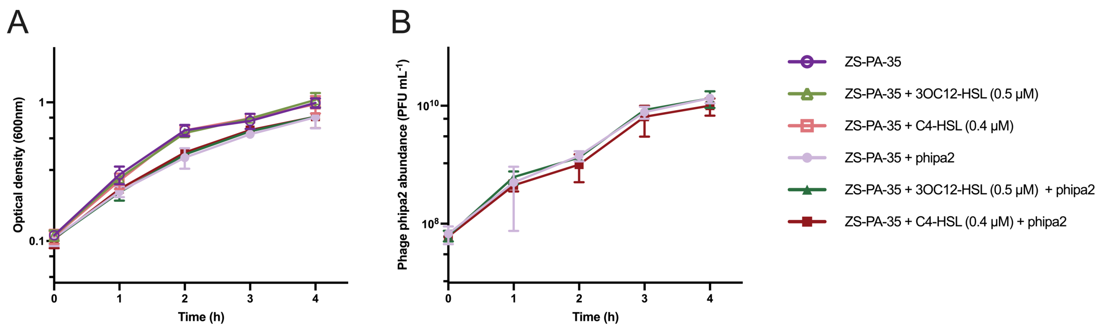

Figure S2. The effects of individual synthetic AI (0.5 μM 3OC12-HSL or 0.4 μM C4-HSL) on phage-host interactions. Phage phipa2 was introduced at an initial OD_{600} of 0.1 and MOIs of 2. Optical densities (A) and phage concentrations (B) were measured at 1-h intervals in LB medium with or without AI addition over a 4-h period of incubation. Statistical significance was determined by two-way ANOVA with Dunnett's multiple comparison test. Error bars represent standard deviations from all experiments carried out in triplicate.

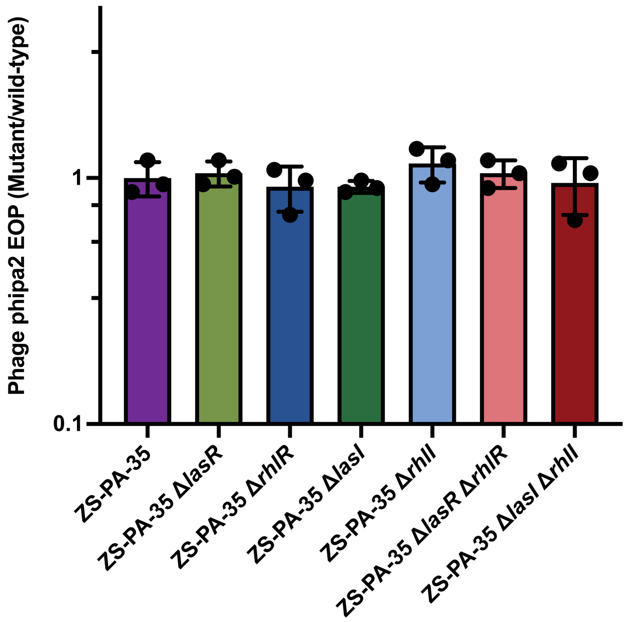

Figure S3. Efficiencies of plating (EOP) were determined by calculating the ratio of plaques formed on each host from phipa2 lysates to those formed on the wild-type strain ZS-PA-35. Statistical analysis was conducted using ordinary one-way ANOVA followed by Dunnett’s test.

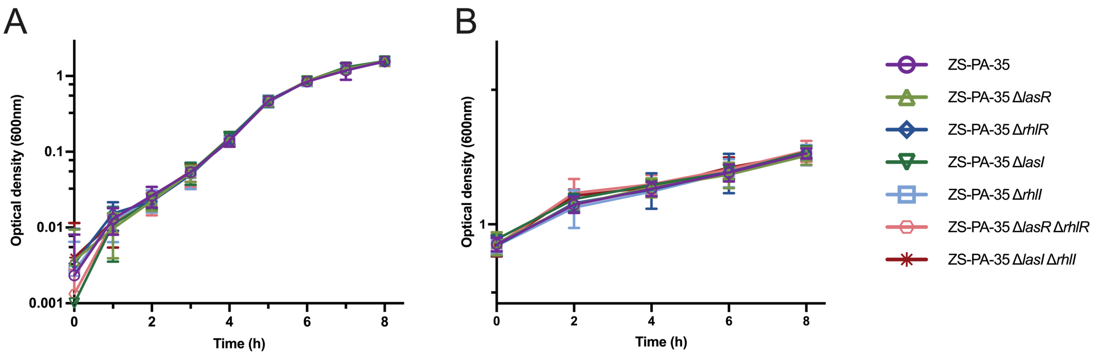

Figure S4. Growth curves of *P. aeruginosa* wild-type (ZS-PA-35) and QS mutants (ZS-PA-35 Δ*lasR*, ZS-PA-35 Δ*rhlR*, ZS-PA-35 Δ*lasI*, ZS-PA-35 Δ*rhlI*, ZS-PA-35 Δ*lasR* Δ*rhlR*, and ZS-PA-35 Δ*lasI* Δ*rhlI*) were measured starting from both LCD (OD_{600} = 0.1) (A) and HCD (OD_{600} = 1.0) (B) cultures over 8 h of incubation, respectively. Data are represented as mean ± SD (error bars). The significance was analyzed by one-way ANOVA with Dunnett's multiple comparison test (n=3).

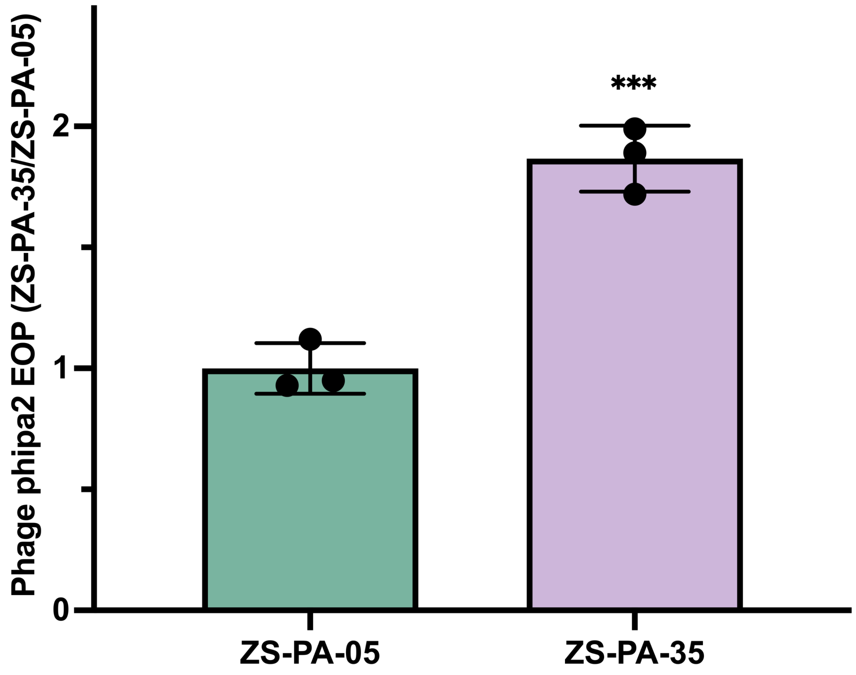

Figure S5. The efficiency of plating (EOP) of phage phipa2 on strains ZS-PA-05 and ZS-PA-35 was assessed by comparing the number of plaques formed on ZS-PA-05 to those formed on ZS-PA-35. Data are presented as mean ± SD. ***P < 0.001, determined by Student's *t*-test (*n* = 3).

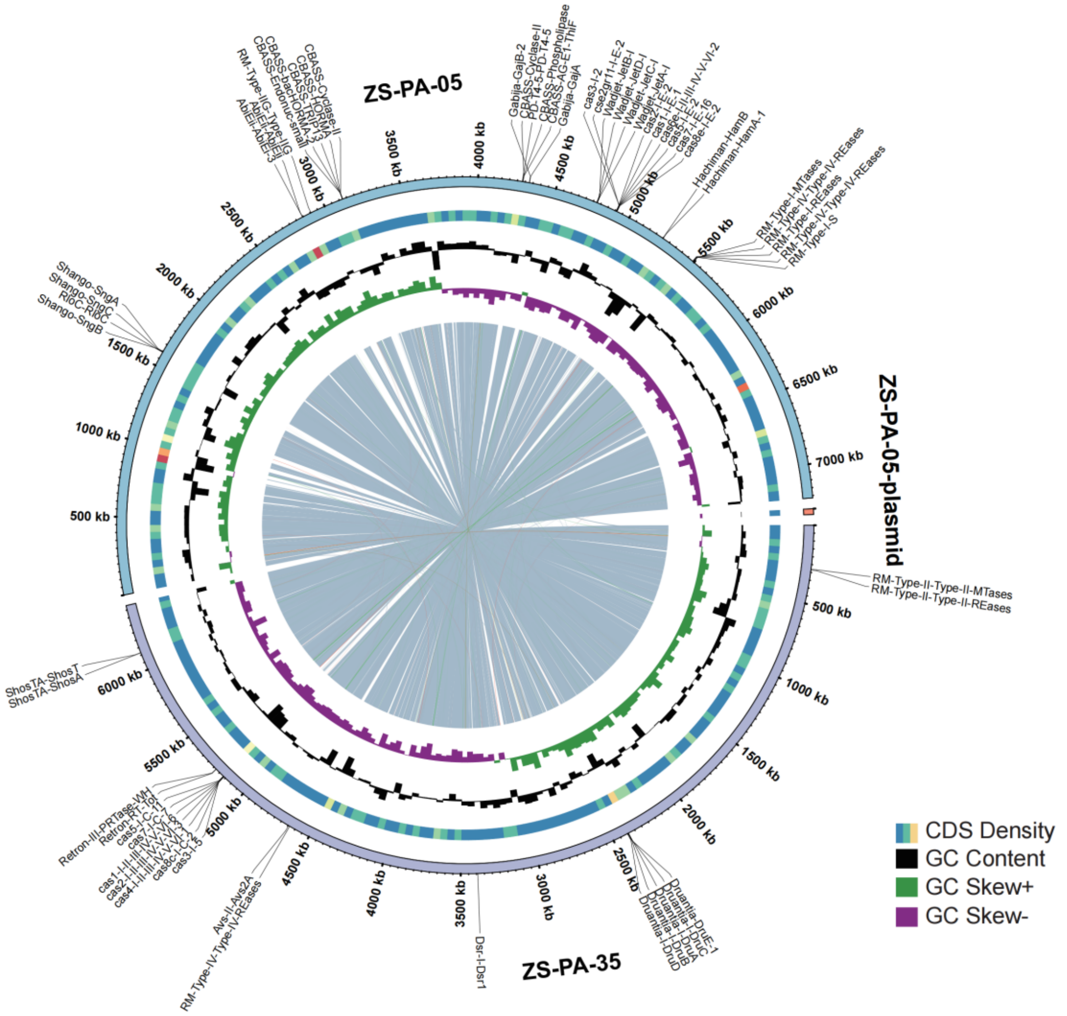

Figure S6. Circos plots visualizing the genomic comparisons between P. aeruginosa strains ZS-PA-05 and ZS-PA-35, with their putative defense systems predicted using the DefenseFinder online tool (https://defensefinder.mdmlab.fr/).

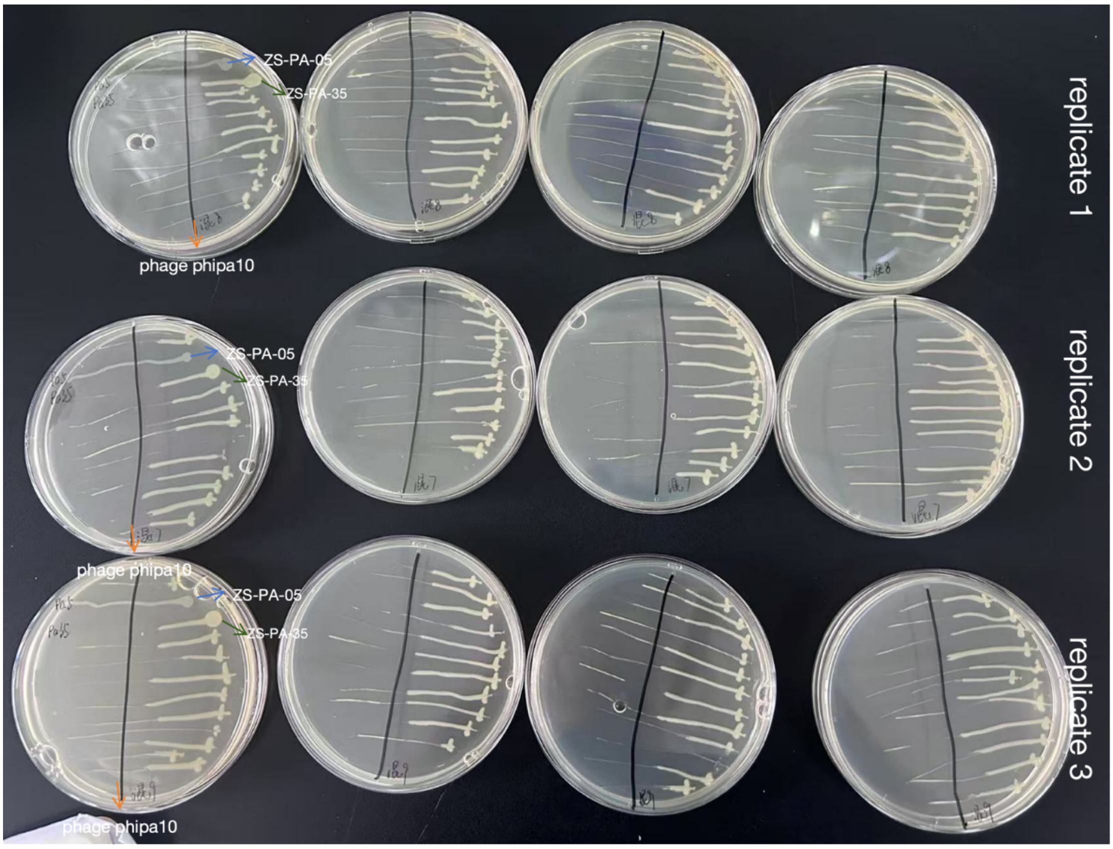

Figure S7. Representative images of the cross-streak assay, performed to analyze the relative abundance of each strain in mixed cultures of *P. aeruginosa* strains ZS-PA-05 and ZS-PA-35 following phage phipa2 infection under rich nutrient conditions. Strains ZS-PA-05 (blue arrow) and ZS-PA-35 (green arrow) were used as controls.
